# Signatures of T and B Cell Development, Functional Responses and PD-1 Upregulation After HCMV Latent Infections and Reactivations in Nod.Rag.Gamma Mice Humanized With Cord Blood CD34^+^ Cells

**DOI:** 10.3389/fimmu.2018.02734

**Published:** 2018-11-22

**Authors:** Sebastian J. Theobald, Sahamoddin Khailaie, Michael Meyer-Hermann, Valery Volk, Henning Olbrich, Simon Danisch, Laura Gerasch, Andreas Schneider, Christian Sinzger, Dirk Schaudien, Stefan Lienenklaus, Peggy Riese, Carlos A. Guzman, Constanca Figueiredo, Constantin von Kaisenberg, Loukia M. Spineli, Stephanie Glaesener, Almut Meyer-Bahlburg, Arnold Ganser, Michael Schmitt, Michael Mach, Martin Messerle, Renata Stripecke

**Affiliations:** ^1^Clinic of Hematology, Hemostasis, Oncology and Stem Cell Transplantation, Hannover Medical School, Hannover, Germany; ^2^Excellence Cluster REBIRTH, Laboratory of Regenerative Immune Therapies Applied, Hannover Medical School, Hannover, Germany; ^3^Partner Site Hannover-Braunschweig, German Center for Infection Research (DZIF), Braunschweig, Germany; ^4^Department of Systems Immunology and Braunschweig Integrated Centre of Systems Biology (BRICS), Helmholtz Centre for Infection Research, Braunschweig, Germany; ^5^Institute for Biochemistry, Biotechnology and Bioinformatics, Technical University Braunschweig, Braunschweig, Germany; ^6^Institute of Virology, University of Ulm, Ulm, Germany; ^7^Fraunhofer Institute for Toxicology and Experimental Medicine (ITEM), Hannover, Germany; ^8^Institute for Laboratory Animal Science, Hannover Medical School, Hannover, Germany; ^9^Department of Vaccinology and Applied Microbiology, Helmholtz Centre for Infection Research Braunschweig, Braunschweig, Germany; ^10^Department of Transfusion Medicine, Hannover Medical School, Hannover, Germany; ^11^Clinic of Gynecology and Obstetrics, Hannover Medical School, Hannover, Germany; ^12^Institute for Biostatistics, Hannover Medical School, Hannover, Germany; ^13^Department of Pediatrics, University Medicine Greifswald, Greifswald, Germany; ^14^Department of Hematology, Oncology and Rheumatology, GMP Core Facility, Heidelberg University Hospital, Heidelberg, Germany; ^15^Institute of Virology, University Erlangen-Nürnberg, Erlangen, Germany; ^16^Institute of Virology, Hannover Medical School, Hannover, Germany

**Keywords:** HCMV, reactivation, humanized mice, T cell maturation, B cell class switch, optical imaging analyses, principal component analyses, linear discriminant analyses

## Abstract

Human cytomegalovirus (HCMV) latency is typically harmless but reactivation can be largely detrimental to immune compromised hosts. We modeled latency and reactivation using a traceable HCMV laboratory strain expressing the *Gaussia* luciferase reporter gene (HCMV/GLuc) in order to interrogate the viral modulatory effects on the human adaptive immunity. Humanized mice with long-term (more than 17 weeks) steady human T and B cell immune reconstitutions were infected with HCMV/GLuc and 7 weeks later were further treated with granulocyte-colony stimulating factor (G-CSF) to induce viral reactivations. Whole body bio-luminescence imaging analyses clearly differentiated mice with latent viral infections vs. reactivations. *Foci* of vigorous viral reactivations were detectable in liver, lymph nodes and salivary glands. The number of viral genome copies in various tissues increased upon reactivations and were detectable in sorted human CD14^+^, CD169^+^, and CD34^+^ cells. Compared with non-infected controls, mice after infections and reactivations showed higher thymopoiesis, systemic expansion of Th, CTL, Treg, and Tfh cells and functional antiviral T cell responses. Latent infections promoted vast development of memory CD4^+^ T cells while reactivations triggered a shift toward effector T cells expressing PD-1. Further, reactivations prompted a marked development of B cells, maturation of IgG^+^ plasma cells, and HCMV-specific antibody responses. Multivariate statistical methods were employed using T and B cell immune phenotypic profiles obtained with cells from several tissues of individual mice. The data was used to identify combinations of markers that could predict an HCMV infection vs. reactivation status. In spleen, but not in lymph nodes, higher frequencies of effector CD4^+^ T cells expressing PD-1 were among the factors most suited to distinguish HCMV reactivations from infections. These results suggest a shift from a T cell dominated immune response during latent infections toward an exhausted T cell phenotype and active humoral immune response upon reactivations. In sum, this novel *in vivo* humanized model combined with advanced analyses highlights a dynamic system clearly specifying the immunological spatial signatures of HCMV latency and reactivations. These signatures can be merged as predictive biomarker clusters that can be applied in the clinical translation of new therapies for the control of HCMV reactivation.

## Introduction

Human cytomegalovirus (HCMV) is a ubiquitous and strict human herpesvirus. Whereas, latency after primary infection is mostly harmless, HCMV reactivations have been known for more than 30 years to increase the non-relapse mortality for patients after allogeneic hematopoietic stem cell transplantation (allo-HSCT) (1–5). HCMV latency invokes complex mechanisms of immune modulation and evasion such as down-regulation of the major histocompatibility complexes (MHCs) (6–8), production of anti-inflammatory viral cytokines (9) and T cell inflation (MHC) (10, 11). HCMV reactivations after allo-HSCT were shown to promote immune suppressive effects on T cells (12–14) and, on the other hand, immune stimulatory effects on B cells (15–17). Despite its ubiquitous and global detrimental effects, vaccines, or immune therapies to protect against HCMV reactivations have not been approved (18). Recapitulating the effects of HCMV reactivation on the immune system in an *in vivo* experimental system could guide toward predictive immunologic patterns for future testing of vaccines and immune therapies in humans.

Due to the strict species-specificity of HCMV, mouse and primate models cannot be used to clarify the spatio-temporal mechanisms associated with HCMV reactivations *in vivo* (19). Pioneering studies by Mocarski et al utilized immune-deficient male NOD-*scid* mice co-implanted with human fetal tissues [thymus (THY), liver (LI), lung, colon and skin], and then, largely varying from 1 to 6 months later, mice were infected with HCMV (Toledo or Towne strains) (20). This system showed *in vivo* tropism of HCMV lytic virus replications in human epithelial cells, latency in hematopoietic cells and confirmed antiviral effects of ganciclovir. Subsequently, Nelson et al. further improved this HCMV infection model using human bone marrow (BM) cells, liver and thymus tissues (also known as BLT model) implanted into different body parts of NOD-*scid* mice (21). BLT xenograft systems are known to show a robust engraftment and early human immune reconstitution in NOD.Cg-*Prkdc*^*scid*^*IL-2R*$\gamma_{c}^{tm1Wjl}$ (NSG) mice (22, 23), but inopportunely, the variable early onset of xenograft GVHD (xeno-GVHD) in this BLT model is frequently lethal (24, 23). NSG/BLT mice were injected i.p. with neonatal human dermal fibroblasts (NHDF) infected with an HCMV and then implanted with mini-pumps for constant human granulocyte-colony stimulating factor (hG-CSF) release. HCMV reactivations were shown as the increase in the numbers of genome viral copies in peripheral blood (PBL), spleen (SPL), LI, and kidney (25). More recently, using a short-term 12 weeks NSG/BLT model of HCMV infection (clinical strain TRpM1A or laboratory strain TB40/GFP), the same group detected human CD4^+^ and CD8^+^ T cell responses against the viral immediate early protein 1 (IE1) and the phosphoprotein pp65 and humoral human responses with the ability to neutralize HCMV *in vitro* (25). Nevertheless, since proper maturation of human T and B cells require at least 15–20 weeks after human stem cell engraftment to occur (26), the experimental design of these humanized mice studies did not allow the evaluation of the T and B cell development. Further, a general major obstacle of this complex NSG/BLT model is the scarcity of fetal tissues for establishment of xenografted mice, currently facing ethical constrains.

In order to bypass all these limitations, we explored an alternative human reconstitution model consisting of NOD.Cg-*Rag1*^*tm*1*Mom*^*IL-2R*$\gamma_{c}^{tm1Wjl}$ (NRG) female mice transplanted with largely available cord blood (CB) purified CD34^+^ cells. In our hands, this robust system showed consistent long-term (20 to more than 30 weeks) development of functional and mature T and B cell responses (with rare incidences of xeno-GVHD) and, when combined with dendritic cell (DC) immunizations, we obtained patterns of predictive phenotypic markers of immune responses (26–29). Here, we interrogated this long-term humanized mouse model whether the immune effects of HCMV primary infections vs. reactivations could be distinguished. We established a new model using an engineered HCMV laboratory strain secreting *Gaussia* luciferase (GLuc) (30) and performed non-invasive optical imaging analyses to follow the bio-distribution and extent of viral infections. HCMV reactivations after G-CSF administrations were confirmed by optical imaging and genome copies analyses in different tissues. Spatial analyses of human T and B cell development generated immune phenotypic data demonstrating a clear divergence between primary infections and reactivations. Reactivations were particularly associated with up-regulation of the programmed cell death (PD)-1 activation marker on T cells and B cell activation. Multivariate statistical analyses such as principal component analyses (PCA) and linear discriminant analyses (LDA) clustered the predictive markers and corroborated the different immunologic signatures reflecting infections and reactivations. The parameters obtained from this predictive and dynamic humanized mouse model system can be implemented to test new drugs, vaccines and immune therapies aimed at controlling HCMV reactivation.

## Methods

### Ethics statements

Umbilical CB units were obtained under written informed consent of the mothers in accordance with study protocols approved by Hannover Medical School Ethics Review Board (approval Nr. 4837). All experiments involving mice were performed in accordance with the regulations and guidelines of the animal welfare of the State of Lower Saxony (“Niedersächsiches Landesamt für Verbraucherschutz und Lebensmittelsicherheit, Dezernat 33/Tierschutz,” approval Nr. 33.12-42502-04-1). Euthanasia was performed with cervical dislocation after CO_2_ narcosis.

### Cell culture and primary cells

MRC-5 (human fetal lung fibroblasts) and HEK-293T cells (ATCC, Manassas, VA, USA) were expanded in DMEM (ThermoFisher, Waltham, MA) supplemented with 10% fetal bovine serum (FBS, HyClone, Logan, UT), non-essential amino acids (ThermoFisher) and penicillin/streptomycin (Merck Millipore, Billerica, MA) at 37°C with 5% CO_2_. Mononuclear cells were isolated by Ficoll gradient centrifugation, CD34^+^ cells were purified after two rounds of immune-magnetic bead positive selection (Miltenyi Biotech, Bergisch-Gladbach, Germany).

### Production and titration of HCMV/GLuc and generation of virus-carrier cells

HCMV/GLuc seeding stocks were generated using a TB40-BAC4-cloned HCMV variant and propagated on MRC-5 cells as described (30). Cell-free supernatants were concentrated by centrifugation at 19,000 rpm for 2 h 20 min, re-suspended in Dulbecco's modified Eagle medium (DMEM, ThermoFisher) plus 1% non-essential amino acids (ThermoFisher) and stored at −80°C. Viral batches were titered in MRC-5 cells by immunofluorescence analyses using the mouse anti-IE1 p63-27 antibody essentially as described (30). For the generation of batches of HCMV-infected carrier cells, 1.0 × 10^7^ MRC-5 cells were infected at a multiplicity of infection (MOI) of 1 overnight at 37°C, the medium was replenished and then the cells were cultivated for additional 2 days. Cells were detached with Trypsin/EDTA (Merck Millipore), centrifuged at 300 g for 10 min, washed with PBS and cryopreserved in freezing medium containing 15.5% human albumin, 10% DMSO (Braun, Melsungen, Germany), and 5% glucose (Sigma-Aldrich, St.Louis, MO). A thawed cell aliquot was stained for detection of gB on the cell surface using the mouse anti-gB p27-287 antibody and a secondary anti-mouse-Alexa647 antibody (Biolegend, San Diego, CA). The frequency of gB-positive cells was determined by flow cytometry using an LSR II cytometer (BD Bioscience, Becton Dickinson GmbH, Heidelberg, Germany).

### Bio-luminescence detection in cell supernatants containing *gaussia* luciferase

Cell culture supernatants were collected and frozen at −80°C. Twenty microliter of thawed supernatant were mixed with 50 μl BioLux® Gaussia Luciferase Assay Kit according to the manufacturer's instructions (NEB, Frankfurt am Main, Germany) or with 50 μl 0.2 μg/ml coelenterazine (Promega, Mannheim, Germany). Bio-luminescence (light intensity per second) was measured using an Orion II Microplate Luminometer (Titertek-Berthold, Pforzheim, Germany) or a Tristar^2^ Microplate Reader (Berthold Technologies, Bad Wildbach, Germany).

### HSCT into NRG mice

NRG mice were obtained from The Jackson Laboratory (JAX, Bar Harbor, ME) and bred in-house under pathogen-free conditions. We used only female NRG mice, as we have shown that they supported better T cell development (28) and differentiation after DC immunizations (26). Prior to HSCT, 5 weeks-old mice were sub-lethally irradiated (450 cGy) using a [137^Cs^] column irradiator (Gammacell 3000 Elan; Best Theratronics, Ottawa, Canada). Four hours after irradiation, 2.0 × 10^5^ human CB-CD34^+^ cells were injected into mice through the tail vein as described (27, 29).

### Infection of humanized mice with HCMV/GLuc

Humanized mice were generated and analyzed essentially as described (26–29). Frequencies of human immune cell subsets in the PBL samples were determined at 17 weeks after HSCT. Next, mice were treated with 150 ng mouse/day s.c. with hG-CSF (Granocyte, Kohlpharma GmbH, Merzig, Germany) for 5 days. On the third day of hG-CSF administration, 1.0 × 10^6^ cryopreserved/thawed viable HCMV-infected MRC-5 cells were injected i.p. 7 weeks after infection, one cohort was treated with hG-CSF for 7 days (2.5 mg/mouse/day s.c.) to stimulate viral reactivations. After optical imaging analyses and euthanasia, several tissues were collected SPL, lymph nodes (LN), mesenteric lymph nodes (mLN), BM (flushed from two femurs), salivary glands (SG), and LI, dissociated into single cell suspensions and the numbers of viable cells per tissue were quantified by trypan blue exclusion assay.

### *In vivo* optical imaging analyses of humanized mice infected with HCMV/GLuc

Mice were anesthetized with Ketamin/Xylazine (10 and 0.5 mg/ml, respectively, Sigma-Aldrich). Coelenterazine (Promega, Madison, WI) was solubilized in ethanol (5 mg/ml) and diluted shortly prior to administration in PBS (final dose 50 μg/mouse). Mice were injected i.v. with 100 μl of coelenterazine solution and imaged immediately. *In vivo* optical imaging analyses were performed with an IVIS 200 optical imaging system (PerkinElmer, Waltham, MA) or with an IVIS SpectrumCT (PerkinElmer). Different CB stem cell sources were used for R1 and R2. Pictures were taken with a field of view C, f stop 1 and medium binning for each mouse. Exposure time was set to 300 s per mouse. The anatomical region of interest (ROI) was kept constant for quantified analyses of all mice.

### FACS analyses

Cells obtained from peripheral blood or tissues were treated with a hypotonic solution (0.83% ammonium chloride/20 mM HEPES, pH 7.2, for 5 min at room temperature, followed by stabilization with cold PBS and washing) in order to remove the erythrocytes and further processed as described as fresh or cryopreserved/thawed material for immune staining and flow cytometry analyses (29, 26). The appropriate concentration of each antibody was determined in the titration assay before the main experiment. After the cells were washed to remove unbounded antibodies and immune fluorescence was assessed by LSR II cytometer (BD Biosciences). Commercially available antibodies against all markers are provided in Table S1.

### Quantitative real-time PCR (RT-q-PCR) for detection of HCMV DNA

Tissues were processed with the Blood and Tissue isolation kit (Qiagen, Hilden, Germany). DNA concentration was determined by spectrophotometry and stored at −20°C. Twenty microliter of the DNA sample was used for PCR analyses with an Artus® CMV TM PCR kit according to the manufacturer's instructions (Qiagen). The standard curve was determined with primers specific for HCMV sequences and internal control primers against a human gene. PCR was performed and analyzed with StepOnePlus-PCR cycler (ThermoFisher). The Ct values were used to calculate the copies of HCMV genomes per sample adjusted with the respective DNA concentrations. For detection of HCMV in human lymphocyte subsets, cells were sorted using a FACS Aria 2 (BD Bioscience).

### Detection of human immunoglobulins and human cytokines in serum

Concentrations of human Th1/Th2 cytokines in serum were quantified by fluorescent bead–based 14-plex Luminex assay (Merck Millipore) according to the manufacturer's instructions. Following human cytokines were analyzed: GM-CSF, IL-4, TNF-α, IL-6, IL-8, MCP-1, IL-10, IL-1b, IL-5, IFN-γ, IL-7, IL-2, and IL-12(p70). The human IgG and IgM ELISA kit (eBioscience, San Diego, CA) was used corresponding to instructions. Serum samples separated from peripheral blood were thawed on ice, diluted, and samples were measured in triplicates. Plates were analyzed with Spectramax 340PC384 plate reader (Molecular Devices, Sunnyvale, CA).

### Immunohistochemistry analyses

Following a fixation period of 24 h in 3.7% formaldehyde buffer (Roth, Karlsruhe, Germany) the spleen was embedded in paraffin. Three micrometer-thick sections were prepared, dewaxed, and subjected to immunohistological staining as described (29). Briefly, heat-induced antigen retrieval was performed in a citrate-buffered solution. All slides were rinsed with Tris-buffered saline (pH 7.6) plus 0.01% Tween®20 (Merck, Darmstadt, Germany). Slides were incubated for 20 min at 21°C in normal goat serum (Vector Laboratories Inc., Burlingame, CA) and then incubated with the primary antibody (anti-human nuclei, CD3 and CD79a) for 1 h at 21°C. Secondary biotin-SP-conjugated antibody was applied for 30 min at 21°C. Final staining was achieved by routine method using alkaline phosphatase streptavidin-biotin (Vector Laboratories Inc., Burlingame, CA) and Fast Red as chromogen (Fast Red substrate pack, BioGenex, Freemont, CA). The slides were counterstained using Bluing Reagent (Thermo Scientific, Braunschweig, Germany). Cover-slipping was done using Aquatex® aqueous mounting medium (Merck KGaA, Darmstadt, Germany). Sample permeabilization, antibody concentrations, antibody reactions, and staining procedures were previously optimized for each antibody (29) to get clear and specific immunohistochemical signals. Slides were elevated using a bright-field microscope. Whole slides were digitized using the Mirax scanner (Zeiss, Oberkochen, Germany) equipped with a 20x plan-apochromat lens (Zeiss) and a camera (Hitachi_HV_F22CL, Hitachi Kokusai Electric Europe, Neu-Isenburg, Germany). Pictures for the figures were taken with the Panoramic Viewer (3dHistech, Budapest, Hungary) as viewing software.

### ELISpot analyses using recombinant HCMV antigenic proteins

BM samples were thawed from individual mice (Exp. 4, Table 1, CTR = 3, INF = 4, REAC = 4), and mononuclear cells were used directly for IFN-γ ELISpot assay using the T-track human Kit according to the vendor's recommendations (Lophius Bioscience, Regensburg, Germany) (31). PBMCs obtained from HCMV sero-positive and sero-negative donors were used as controls. Cell suspensions (2 × 10^5^ cells/ 100 μl) were mixed in duplicates with IE1 or pp65 recombinant proteins (following the vendor's recommendations, Lophius bioscience) or with gB recombinant protein (250 μg/ml, Sino Biologicals, Beijing, China). Subsequently, the mixtures of cells plus antigens were transferred onto ELISpot plates pre-coated with anti-IFN-γ antibodies and incubated for 20 h at 37°C. Immune detection of IFN-γ spots was performed following the vendor's recommendations (Lophius Bioscience). Spots on the wells were photographed and counted using a microscope and the results depicted as spot forming cells (SFC) within 2 × 10^5^ PBMC.

**Table 1:** Immune reconstitution analysis of human lymphocytes in peripheral blood of humanized mice prior to the experimental treatments.

**Experiment**	**huCD45^+^ (%)**	**huCD3^+^inCD45^+^ (%)**	**huCD4^+^inCD45^+^ (%)**	**huCD8^+^inCD45^+^ (%)**
Exp1 (week 15) ±*G*−*CSF*	38.4 ± 10.1	16.1 ± 6.34	6.3 ± 2.3	4.1 ± 3.0
Exp2 (week 17) LV vs. HCMV	52.0 ± 8.0	19.1 ± 3.85	11.8 ± 2.7	6.59 ± 1.5
Exp3/R1(week 17) INF vs. REAC	27.9 ± 14.9	47 ± 10	21.3 ± 9.1	14.1 ± 4.6
Exp4/R2(week 17) INF vs. REAC	43.7 ± 10.4	10.9 ± 2.5	7.8 ± 1.4	4.9 ± 2.0

### ELISpot analyses using HCMV-infected dendritic cells

LN and mLN samples were thawed and pooled (Exp. 4, Table 1, CTR = 3, INF = 4, REAC = 4). The corresponding CB CD34 negative fraction was used to isolate CD14+ monocytes. For the generation of Conventional DCs (conDC), cells were cultured in X-vivo media (Lonza, Basel, Switzerland**)** with recombinant human IL-4 and GM-CSF (both 50 ng/ml; Cellgenix, Freiburg, Germany) for 7 days. For generation of conDC infected with HCMV-GLuc (conDC/HCMV), monocytes cultured with cytokines for 4 days were infected with virus at M.O.I. = 1 and maintained in culture for additional 3 days. Prior to ELISpot, DCs were analyzed by flow cytometry to confirm expression of relevant markers (CD80, CD86; HLA-DR, gB see Table S1). Then, DCs and mononuclear cells (1 × 10^5^ cells each in 100 μl) were co-cultured on the ELISpot plate as described before for 20 h. Spots were counted automatically using an ELISpot reader (AID, Straßberg, Germany) and the results depicted as SFC/1 × 10^5^ PBMC.

### Dot blot analysis for detection of plasma IgG against gB

293T-wt and 293T-gB cells were re-suspended in PBS and maintained on ice. Cell lysates were produced by sonication and the protein concentration was determined with Bicinchoninic acid assay (BCA). One microliter corresponding to 1 μg of cell lysate was spotted onto strips of nitrocellulose membranes (GE Healthcare, Little Chalfont, UK) and air-dried for 10 min. Five percent milk/PBS/0.05% Tween (Merck Millipore) was used to block the membrane for 30 min at room temperature. Plasma from mice was added to the membrane strips which were incubated overnight at 4°C and washed 3x with PBS/0.05% Tween for 10 min at RT. Anti-human IgG-HRP (Roth, Karlsruhe, Germany) (1:500) was added for 1 h at RT. Next, the membrane was washed 4x with PBS/Tween for 10 min at room temperature (RT). After incubation with HRP chemiluminescent substrate (ThermoFisher), signals were measured with a Chemidoc station (Bio-Rad, Hercules, CA). ImageJ software (NIH, freeware) was used for signal quantifications of dot-blots.

### Quantitative ELISA for detection of plasma IgG against gB

Cell lysates were produced as described above from 293T-wt, 293T-gB, MRC-5, and MRC-5 cells infected with HCMV-GLuc (3 days post-infection, MOI = 1). After determination of the protein concentrations, 96 well plates (Coring, Corning, NY) were coated with 640 ng/50 μl of each cell lysate. The plates were incubated overnight at 4°C and washed 5x with PBS/0.1%Tween. The wells were pre-blocked by replacing the lysates with 100 μl of PBS/5% FBS in a wet chamber for 2 h at 37°C and then washed 3X with PBS/0.1% Tween. As a reference to validate the quantification obtained with the ELISA measurements we used 50 μl a human purified SM5-1 monoclonal antibody that was serially diluted up to 1 × 10^−4^ ng/ml and applied to wells coated with the 293T-gB lysate. The plasma samples from individual mice were diluted at 1:10 (in PBS/2% FBS) and applied to the wells coated with the different cell lysates. The plasma samples from each mouse were analyzed in duplicates for each cell lysate. The plates were incubated for 2 h at 37°C, washed 5x with PBS/0.1% Tween and then incubated with anti-human IgG-HRP (1:1,000) for 45 min at 37°C. After washing 3x with PBS/0.1% Tween, 100 μl of the TMB solution (ThermoFisher) were added to each well and reaction was stopped with 100 μl H_2_SO_4_ (Roth). The OD450 was measured in an ELISA plate reader.

### Statistical analyses

Data consisting of the percentage of human lymphocyte subsets in blood and tissues and the absolute counts of different human lymphocyte subsets in tissues (# of positive cells and % positive) were organized in a PivotTable using Excel software 2010 (Microsoft, Redmond, WA). Each sample was coded according to the reconstitutions (R1 or R2), cohorts (CTR, INF, and REAC), mice IDs, time-point of analyses, tissues, and lymphocyte subtypes. Negative binomial regression was used to compare the cohorts in terms of total cell counts of lymphocyte subtypes in tissues using rate ratio, whereas beta regression was applied to compare the cohorts regarding the percentage of human lymphocyte subsets in blood and tissues using odds ratio. One-way analysis of variance (ANOVA) was performed for statistical analyses to differentiate the cohorts regarding the measurement of bio-luminescence signals after optical imaging analyses, human cytokines in plasma, human immune globulins in plasma, frequencies, and absolute counts of Tregs and Tfh cells and the determination of viral neutralization *in vitro*. Parameter estimation was performed by least square means. *P*-values were calculated at significance levels 0.05 using two-sided *t*-test statistical analyses (estimated *p*-values lower than 0.01 have been indicated). All analyses were implemented using the SAS 9.3 software (SAS, Cary, NC). PROC GENMOD and PROC GLIMMIX procedures were used for the negative binomial and beta regression analysis, respectively. GraphPad Prism version 5 software (GraphPad Software, Inc., La Jolla, CA) was used to create all figures.

### Principal component and linear discriminant analyses

Principal component analysis (PCA) is a multivariate statistical technique used to simplify high-dimensional data by emphasizing the data variations. PCA expresses the underlying information in data with a new set of variables called principal components (PC). PCs specify directions where the data is widely distributed and a significant proportion of data variability can be explained. By calculating the correlation of original data with the PCs, one can identify the markers with the highest correlation/anti-correlation value. We employed PCA in our experimental data to identify a subset of markers accounted for data variations. The relationship between the identified markers was obtained by calculating their correlations. Our analysis consisted of the following steps: (1) variables (markers) were standardized, i.e., normalized with 0 mean value and unit variance; (2) PCA was performed on different subsets of experimental data according to the unit [absolute (#)vs. frequency (%)], cell-type (T-cell vs. B-cell associated markers), and organ (LN, SPL, BM, single-organ/multi-organs); (3) most important T cell and B cell associated markers with highest maximum correlation with the first 3 PCs were selected; (4) correlations between markers were calculated and presented in the form of heat-maps. In order to find the PCs that could distinguish individuals according to their group (control, after HCMV infections, or reactivations), we searched for PCs (among top 5 PCs) that could separate the distribution of samples of distinct groups. Then, the markers with highest contribution to the composition of the separating PCs were obtained as the potential predictors of the group.

Linear Discriminant analysis (LDA) is a multivariate statistical technique for data profiling and clustering which aims to find a linear combination of variables that best separates samples of distinct groups. LDA creates discriminant functions maximizing the “between-class” separability while minimizing the “within-class” variability. We employed LDA on the experimental data to identify top markers that are contributing in the separability of groups. The most important markers for group discrimination were identified according to the contribution of markers in the composition of discriminant functions (the absolute value of associated coefficient). PCA and LDA were performed with 23 observations (7 CTR, 8 INF, 8 REAC) with 130 phenotypic markers (65 absolute numbers and 65 frequencies) using FactoMineR (32) and MASS (33) libraries of the statistical computing software R (version 3.4.3) (34).

## Results

### huNRG mice show reproducible and robust HCMV-GLuc infections and reactivations

**Figure 1:**
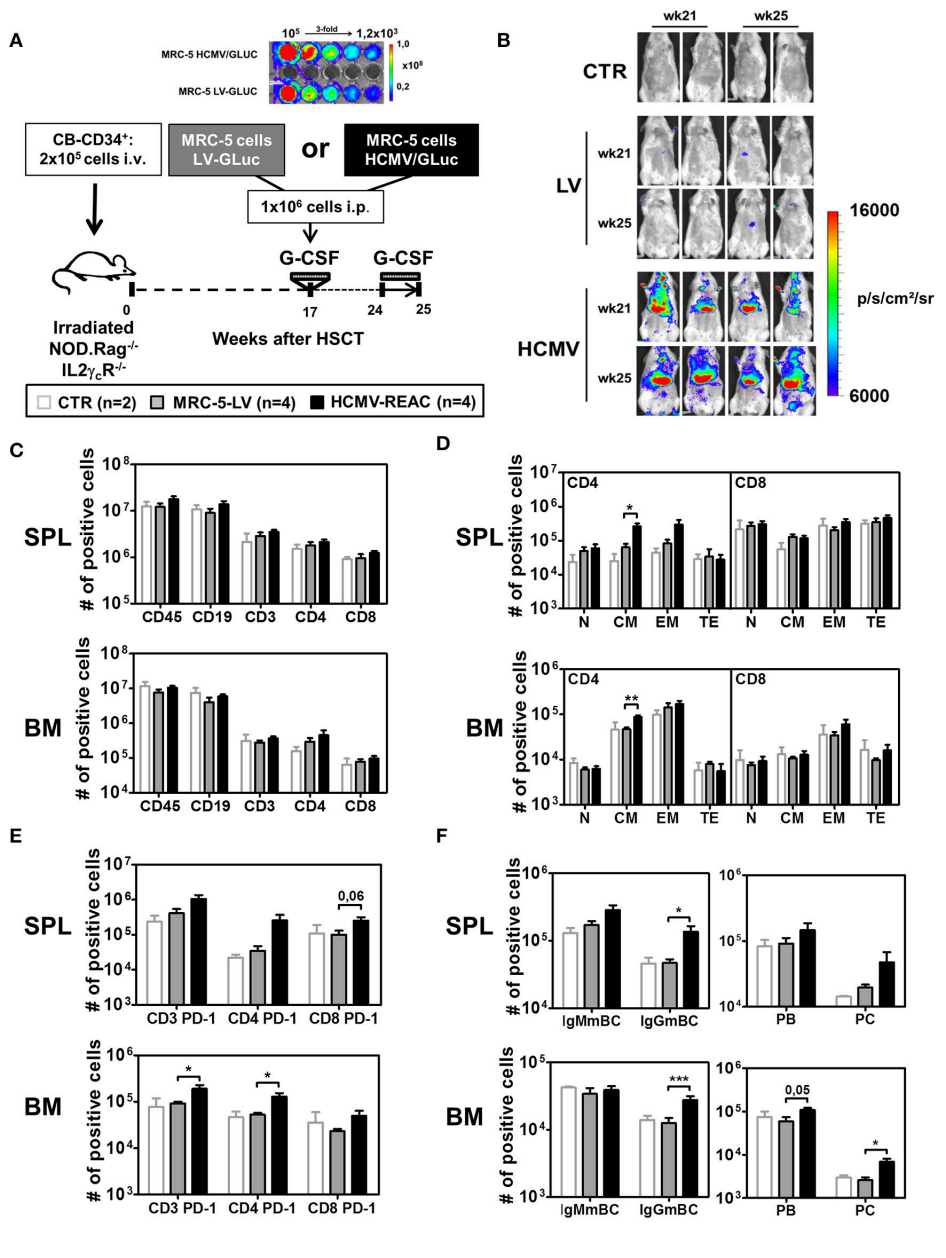
Infection of NRG mice with mock MRC-5 and HCMV-MRC-5 cells followed by stimulation with hG-CSF. **(A)** Schematic representation of experiment 2. MRC-5 transduced with LV-GLuc and MRC-5 infected with HCMV-GLuc showing comparable bio-luminescence signal by optical imaging analyses. As a control group (CTR), irradiated NOD.Rag^−/−^IL2γ_c_R^−/−^ (NRG) mice were transplanted with CD34^+^ CB-HSCT and maintained for 25 weeks. At week 17 after HSCT, experimental groups were treated with hG-CSF (150 ng/mouse/day s.c.) for 5 days and injected i.p. with 1 × 10^6^ MRC-5-LV-GLuc cells (LV) or with MRC-5-HCMV-GLuc cells (HCMV); at week 24 after HSCT they were treated for 7 days with hG-CSF (2.5 μg/mouse/day s.c.). **(B)** Optical imaging analyses performed at weeks 21 and 25 after HSCT showing bio-luminescence signals on different body parts of MRC-5-HCMV-GLuc injected mice (scale bar depicts the average radiance as p/s/cm^2^/sr). **(C)** Quantified total cell numbers (# of positive cells) of human cells recovered from SPL and BM: huCD45^+^ cells within all lymphocytes or CD19^+^ B cells, CD3^+^, CD4^+^, and CD8^+^ T cells within huCD45^+^ cells. Bar graphs represent CTR (white bars), LV (gray bars), and HCMV (black bars) mice. **(D)** Total cell numbers (# of positive cells) are shown for CD4^+^ T cells (left) and CD8^+^ T cells (right) for different subtypes (N, CM, EM, TE) for SPL (top) and BM (bottom). **(E)** Total cell numbers (# of positive cells) for PD-1 expression on CD3^+^, CD4^+^, and CD8^+^ T cells in SPL (top) and BM (bottom). **(F)** Analysis of B cell maturation showing IgMmBC, IgGmBC (left, IgM^+^, and IgG^+^ memory B cells), PB, and PC (right, plasmablasts, and plasma cells) for SPL (top) and BM (bottom). Statistics were performed with unpaired *T*-test comparing MRC-5-LV and HCMV-REAC (* < 0.05; ** < 0.01; *** < 0.001).

We explored the use of the novel HCMV/GLuc strain expressing the *Gaussia* luciferase reporter gene downstream of the IE1 promoter (30). This promoter is strongly active immediately after HCMV infection, and thus IE1 expression is suitable for determining HCMV infection in several types of cells, including peripheral blood mononuclear cells (PBMCs) (Figure S1A). A high correlation between inoculation dose and bio-luminescence signal was measured 1 day post-infection of MRC-5 fibroblasts with the HCMV/GLuc at MOIs 10^−4^ to 10^2^ (Figure S1B). Cryopreserved batches of HCMV/GLuc-infected MRC-5 cells were produced and, after thawing, flow cytometry analyses showed that 70–90% of the viable cells expressed the HCMV glycoprotein B (gB) on the cell surface (Figures S1C,D). G-CSF is known to mobilize and activate stem cells and myeloid precursors, thereby promoting HCMV infection. In pilot experiments we evaluated the immune modulatory effects of hG-CSF on mice transplanted with human CD34^+^ cells (Figure 1). Importantly, we utilized only banked and cryopreserved CD34^+^ purified cells pre-tested in couple of transplanted NRG mice showing robust long-term (>15 weeks) human reconstitutions. In our laboratory, a sufficient “humanization” is arbitrarily defined by the presence of >20% human CD45^+^ cells within all PBMCs measured at 15 weeks after CB-HSCT or prior to the experimental start. Seventeen weeks after HSCTs, human cell frequencies analyzed in PBL for the four different experiments described below varied: 28–52% huCD45^+^; 11–47% huCD3^+^ in huCD45^+^; 6–21% huCD4^+^ in huCD45^+^, and 4–14% huCD8^+^ in huCD45^+^ (Table 1). Despite this variability among the different experiments, these values were within our expected ranges. At 17 weeks after HSCT, humanized mice were administered subcutaneously and daily for 5 days with a low dose of hG-CSF (150 ng). Twenty-four weeks after HSCT, prior to experimental termination, mice were administered with a high dose for 7 days (2.5 μg/mouse/day) (Figure S1E). Flow cytometry analyses of CD45^+^, CD19^+^, CD3^+^, CD4^+^, CD8^+^ cells performed at 25 weeks post-HSCT showed that the hG-CSF treatment schedule on its own did not affect the development or expansion of human lymphocytes in SPL or BM when compared to transplantation only controls (CTR) (Figure S1F).

Next, we used the same hG-CSF treatment schedule but combined it with HCMV infections (reconstitution data analyzed for PBL shown in Table 1, Exp 2). We established a cryopreserved stock of MRC-5 cells infected with a lentiviral vector (LV) expressing GLuc (MRC5-LV) and a cryopreserved stock of MRC-5 cells infected with HCMV/GLuc. After thawing and serially diluting the cell stocks in a 96 well plate, we confirmed equivalent levels of bio-luminescence by optical imaging analyses (Figure 1A). Humanized NRG mice at 17 weeks after CD34^+^ HSCT were administered with 1x10^6^ MRC-5-LV cells (*n* = 4) and then treated with hG-CSF daily from weeks 24 to 25. These mice showed only background bio-luminescence signals detectable on weeks 21 and 25, similar to the transplantation only controls (CTR, *n* = 2) (Figure 1B). This indicated that the MRC-5 cell did not engraft in the mice and basically disappeared. In contrast, all mice injected with 1 × 10^6^ HCMV/GLuc-infected MRC-5 cells (HCMV-REAC, *n* = 4) showed conspicuous bio-luminescence signals in the ventral anatomical regions, indicating ongoing viral reactivations (Figure 1B). Compared to the MRC-5-LV cohort, mice with HCMV reactivations exhibited higher absolute numbers of CD3^+^, CD4^+^, and CD8^+^ T cells in SPL and BM (Figure 1C, see gating strategy in Figure S2A). We further defined the CD3^+^ T cell maturation subtypes based on the expression of CD62L and CD45RA as previously described (35): naïve (N, 62L^+^, and RA^+^), central memory (CM, 62L^+^, and RA^−^), effector memory (EM, 62L^−^, and RA^−^), and terminal effector (TE, 62L^−^, and RA^+^) (see gating strategy in Figure S2B). Upon HCMV reactivations, the immune phenotype profiles of the CD4^+^ T cells from SPL and BM showed a pronounced elevation in the absolute numbers of central memory (CM) and effector memory (EM) cells (Figure 1D, S1G). Additional examination of T cells in these tissues through expression analyses of PD-1 revealed that HCMV reactivations stimulated both CD4^+^ and CD8^+^ T cell activations Figure 1E, S1G. Remarkably, analyses of B cell markers indicative of class switch showed that HCMV reactivations were associated with major rises in the numbers of mature IgG^+^ memory B cells (IgGmBC), plasma blasts (PB, CD138^neg^), and plasma cells (PC, CD138^pos^) (Figure 1F, Figure S1H) (see gating strategy in Figures S2C,D). Thus, collectively, this data established the set-up and feasibility of the model recapitulating HCMV/GLuc reactivations, introduced the usage of spatial optical imaging analyses to track viral bio-distribution and showed associations between the HCMV reactivations with T and B cell activations.

**Figure 2:**
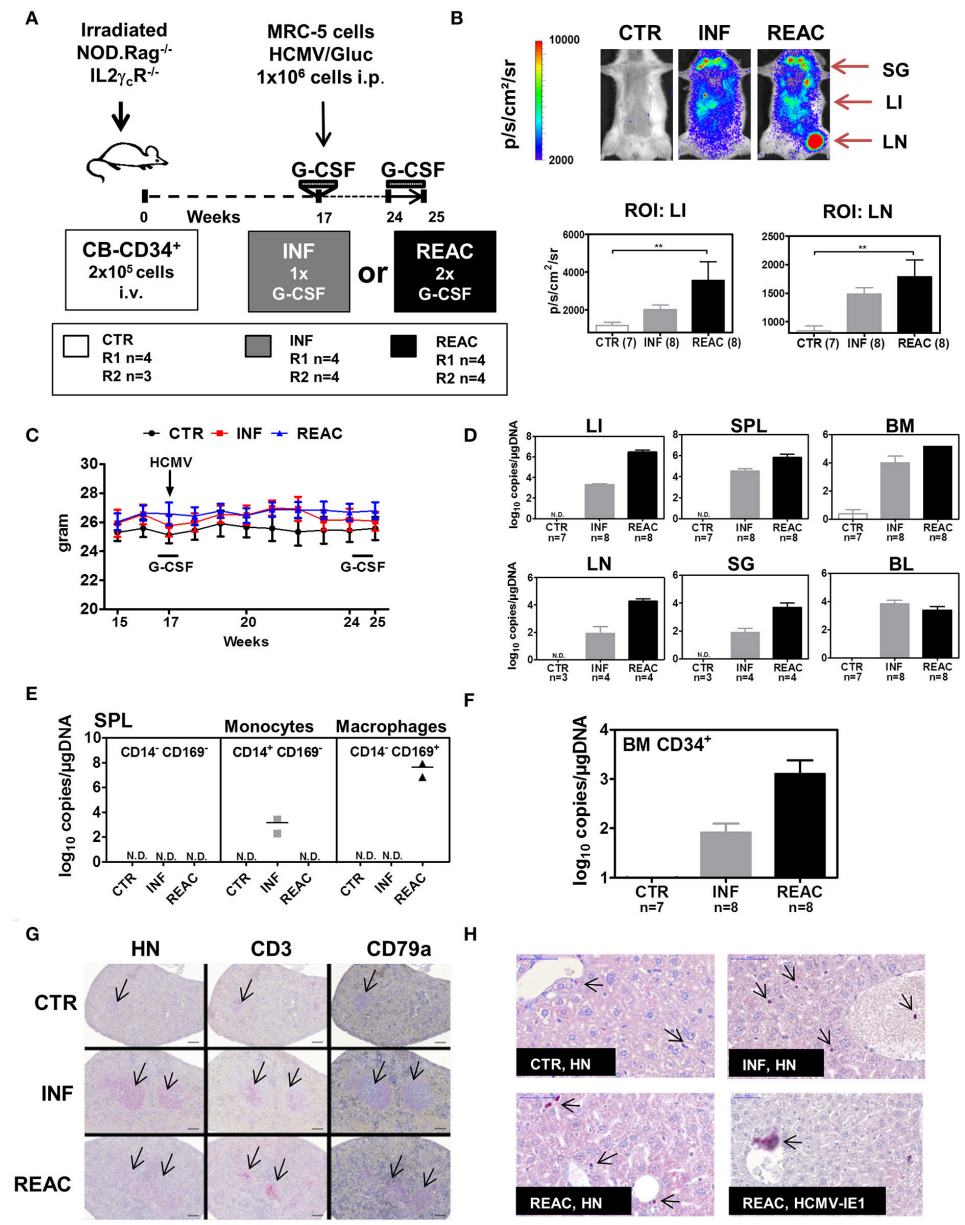
Infections of humanized mice with the HCMV/GLuc strain followed by reactivations stimulated by hG-CSF. **(A)** Schematic representation of experiments 3 and 4. CB-HSCT was performed for generation of control mice (CTR, *n* = 7) using 2.0 × 10^5^ CD34^+^ cells injected i.v. into NRG mice. For the infection group (INF, *n* = 8) 1 × 10^6^ MRC-5 cells infected with HCMV/GLuc were injected i.p. and received 150 ng hG-CSF administered s.c. for 5 days, at week 17. For the reactivation cohort, (REAC, *n* = 8), 2.5 μg hG-CSF were administered s.c. daily for 7 days after week 24. **(B)** Representative pictures obtained from optical imaging analysis for each group (scale bar depicts the average radiance as p/s/cm^2^/sr) and quantified data analyzed by applying the same region of interest (ROI) for each mouse. Statistical analyses were performed with one-way ANOVA (** < 0.01). **(C)** Weekly weight monitoring of mice for each cohort from week 15 to 25 after HSCT for CTR (black), INF (red) REAC (blue). **(D)** RT-q-PCR analysis for detection of HCMV DNA in LI, SPL, BM, LN, SG, and PBL depicted as log_10_ copies/μg DNA. Cohort sizes (*n*) analyzed for each tissue are shown and no detected (N.D.) HCMV DNA for CTR mice. **(E)** Cells from SPL of two mice per cohort were sorted for selection of CD14^+^CD169^−^ monocytes, CD14^−^CD169^+^ macrophages, or the double negative CD14^−^CD169^−^ fraction. HCMV DNA copies were detected by RT-q-PCR and results for each cohort are shown. **(F)** CD34^+^ cells were sorted from BM samples. HCMV DNA copies were detected by RT-q-PCR and results for each cohort are shown. **(G)** Immuno-histopathological analysis of spleen for detection of human cell nuclei (anti-HN), T cells (anti-CD3), and B cells (anti-CD79a). The arrows point to small follicles in representative CTR mouse and enlarged follicles for INF and REAC representative mice. **(H)** Immuno-histopathological analyses of LI. Arrows point to stained human cell nuclei (HN) for representative CTR, INF, and REAC mice. LI of a REAC mouse with an infected cell expressing HCMV-IE1. Size bars at upper left corners correspond to 50 μm.

For the subsequent experiments with larger mice cohorts for in-depth immunological analyses, we compared the effects of HCMV reactivations with primary latent infections. Thus, in addition to the G-CSF double treatment schedule (REAC, *n* = 8), we included one latent infection cohort which did not receive the second hG-CSF daily administrations between weeks 24 and 25 (INF, *n* = 8) (Figure 2A). Mice transplanted with HSCs but not treated were used as baseline control (CTR, *n* = 7). Experiments were performed in duplicate with mice reconstituted with CD34^+^ cells obtained from two different CB donors (Table 1, Exp 3/R1 and Exp 3/R2). Optical imaging analysis performed prior to euthanasia demonstrated high bio-luminescence signals after HCMV infections, which increased after reactivations (Figure 2B). The bio-distribution of the bio-luminescence ranged from speckled signals in the abdominal region to intense spots in the anatomical regions of SG, LI, and LN (Figure 2B). No major changes in body weight were observed after primary HCMV infections or after reactivations (Figure 2C), and no signs of xeno-GVHD were observed macroscopically. HCMV viral DNA was detected in LI of all infected mice (average 10^4^ viral copies/μg DNA), which increased substantially after reactivations (average 10^6^ viral copies/μg DNA) (Figure 2D). This increase was also observed in SPL and BM, but was less pronounced. Further confirming the optical imaging bio-distribution analyses, HCMV DNA copies were detectable in LN and SG of some of the mice and at higher levels after HCMV reactivations (Figure 2D). Remarkably, although all mice were infected, HCMV DNA was inconsistently detected in PBMCs of blood (PBL), i.e., in only about half of the mice after infections and reactivations (Figure 2D). We were able to detect HCMV DNA only in monocytes (CD45^+^/CD14^+^) sorted from spleen after primary infections (INF) but not after reactivations (Figure 2E). On the other hand, we detected HCMV DNA in sorted macrophages (CD45^+^/CD169^+^) after reactivations (REAC), but not after primary infections (INF) only (Figure 2E). CD34^+^ cells, known to become latently infected with HCMV (36), also showed HCMV DNA copies after infections, which increased upon reactivations (Figure 2F). Thus, whereas analyses of BL were not accurate (i.e., showing frequently false negatives values), viral copy numbers in LI, BM, SPL, LN, and SG and in sorted CD34^+^ cells and monocytes were consistently higher after reactivations. Histology analyses revealed that the spleens of infected and reactivated mice were enlarged with clear follicle formations containing human T (CD3^+^) and B (CD79a^+^) cells (Figure 2G). Immune histochemical analyses of LI for detection of human nuclei (HN) showed higher rates of human cells in infected and reactivated mice than in controls, but the occurrence of HCMV/IE1^+^ cells was rare (Figure 2H). All together, these results demonstrated robust qualitative and quantitative differences between HCMV infections and reactivations in this humanized mouse model, providing in addition comprehensible bio-distribution information by non-invasive imaging analyses.

### Human T cells and cytokines in blood are moderately modulated by HCMV

**Figure 3:**
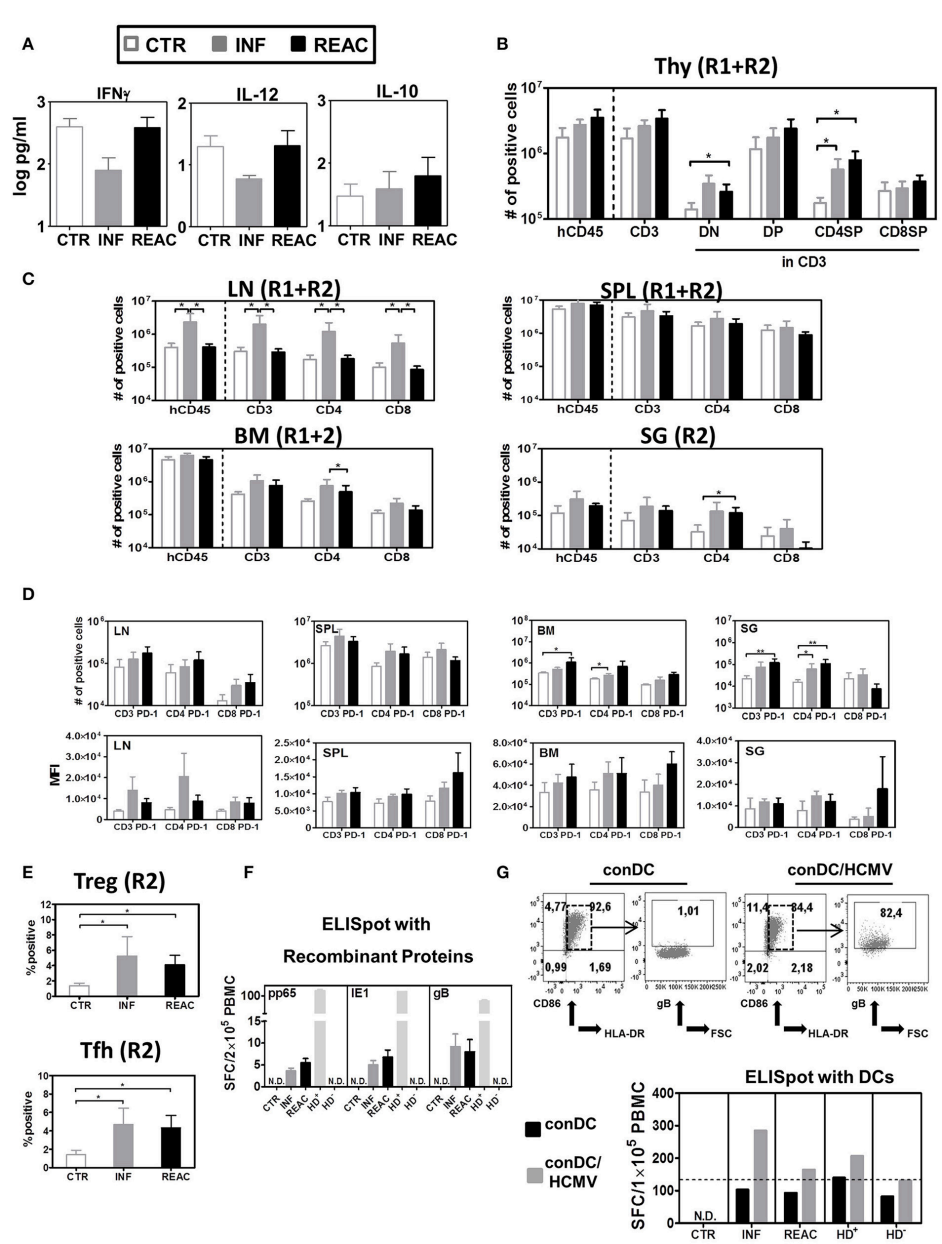
Detection of human cytokines, T cell distribution in different lymphatic and non-lymphatic tissues plus, TREGs, T-FH and functional T cell responses against HCMV. **(A)** Luminex bead-array obtained concentration (pg/ml) of human IFN-γ, IL-12, and IL-10 in mouse plasma. **(B)** Quantified total cell numbers (# of positive cells) of huCD45^+^ cells within all lymphocytes, CD3^+^ T cells within huCD45^+^, and DN, DP, CD4SP, and CD8SP within CD3^+^ T cells. **(C)** Quantified total cell numbers (# of positive cells) of huCD45^+^ cells within all lymphocytes, CD3^+^ T cells within huCD45^+^, and CD4^+^ and CD8^+^ within CD45^+^ T cells for SPL, LN, BM, and SG. Bar graphs represent CTR (white bars), INF (gray bars), and reactivated (REAC, black bars) mice. Reconstitutions included in analysis are indicated (R1+R2: CTR *n* = 7, INF *n* = 8, REAC *n* = 8; R2: CTR *n* = 3, INF *n* = 4, REAC *n* = 4). Error bars represent standard error of the mean. Negative binomial-regression model was applied for statistical analysis (* < 0.05). **(D)** Total cell numbers (# of positive cells) (top panel) and mean fluorescent intensity (MFI, bottom panel) for PD-1 expression on CD3^+^, CD4^+^, and CD8^+^ T cells in SPL (R1+R2), LN (R2), BM (R2), and SG (R2). Negative binomal-regression model was applied for statistical analysis (* < 0.05; ** < 0.01). **(E)** Top Panel: Percentage of FoxP3^+^/CD45RA^−^ regulatory T cells; lower panel: Percentage of PD-1^+^/CXCR5^+^ FTh cells in SPL of individual mice analyzed in R2 corresponding to CTR (white dots), INF (gray dots), and REAC (black dots). One-way ANOVA with Turkey test was used for statistical analysis (* < 0.05). **(F)** IFN-γ ELISpot assay performed with cells recovered from BM (Exp.4) stimulated with proteins. BM samples from mice in each cohort (CTR *n* = 3, INF *n* = 3 and REAC *n* = 3) were stimulated in duplicates with HCMV pp65, HCMV IE1 and HCMV gB recombinant proteins. N.D. depicts non-detectable spots. An HCMV sero-positive donor (HD^+^) and a sero-negative (HD^−^) were used as references. Results are depicted in spot-forming cells (SFC) per 2 × 10^5^ PBMC. **(G)** IFN-γ ELISpot assay performed with cells recovered from LN and mLN (Exp.4) were stimulated with HCMV-infected dendritic cells. Upper panels: Flow cytometry analyses of conventional DCs (conDC) or HCMV-infected conDC (con/HCMV) showing gB expression in the later. Lower panel: Pooled samples obtained from mice in each cohort were co-incubated with conDC or conDC/HCMV in duplicates. N.D. depicts non-detectable spots. HD^+^ and HD^−^ were used as references. Results are depicted in SFC per 1 × 10^5^ PBMC and bar graphs represent the mean values for duplicates.

We next tried to detect immune effects of HCMV infections and reactivations in BL samples that were obtained longitudinally. Whereas, there was only a slight decline in hCD45^+^ frequencies in the CTR and INF groups in the period between 17 and 25 weeks (to 30%), a prominent drop was detectable after reactivations (to 15%) (data did not reach statistical significance) (Figure S3A). But other than that, the relative frequency of B cells, CD4^+^ T, and CD8^+^ T within huCD45^+^ PBL were not much different among the experimental groups (Figures S3B–D). Regarding the T cell maturation subtypes in blood, only the relative frequencies of CD4^+^ and CD8^+^ CM T cells were moderately increased after primary infections, but this effect disappeared after subsequent reactivations (Figures S3E,F). Analyses of human cytokines in plasma revealed that, compared with the other groups, infected mice contained lower concentrations of two important Th1-type cytokines, namely IFN-γ (INF Vs. CTR *p* = 0.07 and INF Vs. REAC *p* = 0.11) and IL-12 (n.s.). On the other hand, analyses of IL-10, which is a typical Th2-type cytokine, showed a moderate increase after HCMV infections, but more so after reactivations (n.s.) (Figure 3A, Table S2). Thus, overall, the HCMV immune modulatory effects observed in circulating lymphocytes and plasma of humanized mice were mild.

### HCMV primary infections and reactivations boost T development in thymus and T cell expansion in lymphatic and infected tissues

We next examined the effects of HCMV infections and reactivations on the T and B cell lineages in primary and secondary lymphatic tissues and several organs for comparative analysis. For comparative analyses, we mostly adopted the absolute human lymphocytes counts, since we showed previously that they were more suitable than relative cell frequencies to reliably characterize immune modulations (26). For thymus, HCMV infections and reactivations stimulated higher hCD45 cellularity and higher absolute numbers of CD4/CD8 double negative (DN, REAC vs. CTR *p* = 0.02), double positive (DP, n.s.), and CD4 single positive (CD4SP, INF vs. CTR *p* = 0.01, REAC vs. CTR *p* = 0.01) T cells (Figure 3B, Table S3). For LN, significantly increased numbers of hCD45^+^ cells (INF Vs. CTR *p* = 0.04, INF Vs. REAC *p* = 0.03), CD3^+^ (INF Vs. CTR *p* = 0.03, INF Vs. REAC *p* = 0.02), CD4^+^ (INF Vs. CTR *p* = 0.03, INF Vs. REAC *p* = 0.03), and CD8^+^ T cells (INF Vs. CTR *p* = 0.04, INF Vs. REAC *p* = 0.02) were observed after HCMV infections compared with the other groups. Similar trends were observed for SPL, BM, and SG (Figure 3C, Table S4). After HCMV infections, and more markedly after reactivations, the numbers of PD-1^+^ T cells were consistently increased for several tissues (Figure 3D: LN, BM, SG; Figure S3: LI and mLN; Table S5). Noteworthy, HCMV infections and reactivations also promoted significantly higher frequencies of CD4^+^/CD25^+^/FoxP3^+^/CD45RA^−^ regulatory T cells (Tregs) (INF Vs. CTR *p* < 0.05, REAC Vs. CTR *p* < 0.05) and CD4^+^/PD-1^+^/CXCR5^+^ T follicular helper cells (Tfh) (INF Vs. CTR *p* < 0.05, REAC Vs. CTR *p* < 0.05) in SPL (Figure 3E). In order to check for functional T cell responses against HCMV antigens, lymphocytes recovered from bone marrow were assayed by IFN-γ ELISpot using pp65, IE1, and gB recombinant proteins as antigens (Figure 3F). Blood cells of healthy donors known to be HCMV seropositive (HD^+^) or seronegative (HD^−^) were used as controls to validate the assay. No reactive spots could be detected with cells retrieved from CTR mice (*n* = 3), whereas T cells recovered from mice after HCMV infections (*n* = 3) and reactivations (*n* = 3) showed detectable spots. Since the detectable number of SFCs was very low using this antigen-specific assay (in the range of 3–12 SFCs per 2 × 10^5^ PBMCs) and we could not differentiate if the responses in mice undergoing HCMV infection or reactivation were distinguishable, we developed another more robust ELISpot assay. We had previously shown that conventional dendritic cells (conDCs) obtained upon culture of monocytes with GM-CSF and IL-4 could be efficiently infected with HCMV laboratory strains (37). Therefore, we generated conDCs matched with the CB CD34^+^ cells used to humanize the mice and infected them with HCMV/GLuc (Figure 3G; as control for the assay, we used non-infected conDCs). Mononuclear cells present in pooled LN and mLN samples (CTR = 3, INF = 4, REAC = 4) were co-cultured with the HCMV-infected or non-infected conDCs on the ELISpot plate for 20 h. Background reactivity due to homeostatic stimulation was detectable for all co-cultures with conDCs. Nevertheless, comparing the number of spots observed when the assay was performed with conDC/HCMV and using effector cells from mice undergoing HCMV infections, we obtained almost 300 SFCs per 1 × 10^5^ PBMCs, and these numbers were half as high for mice undergoing reactivations. Thus, the results obtained with this ELISpot assay seem to provide an inverse correlation between the elevated numbers of PD-1^+^ T cells after HCMV reactivations with lower T cell responses against the HCMV-gB antigen. Since the T cells that develop in these humanize mice are not expected to be restricted to the human HLA, the use of tetramer analyses to verify the PD-1 upregulation on HCMV-specific T cells cannot be explored.

### Reactivations promote the development of memory and terminal effector T cells expressing PD-1 in spleen, but not in lymph node

**Figure 4:**
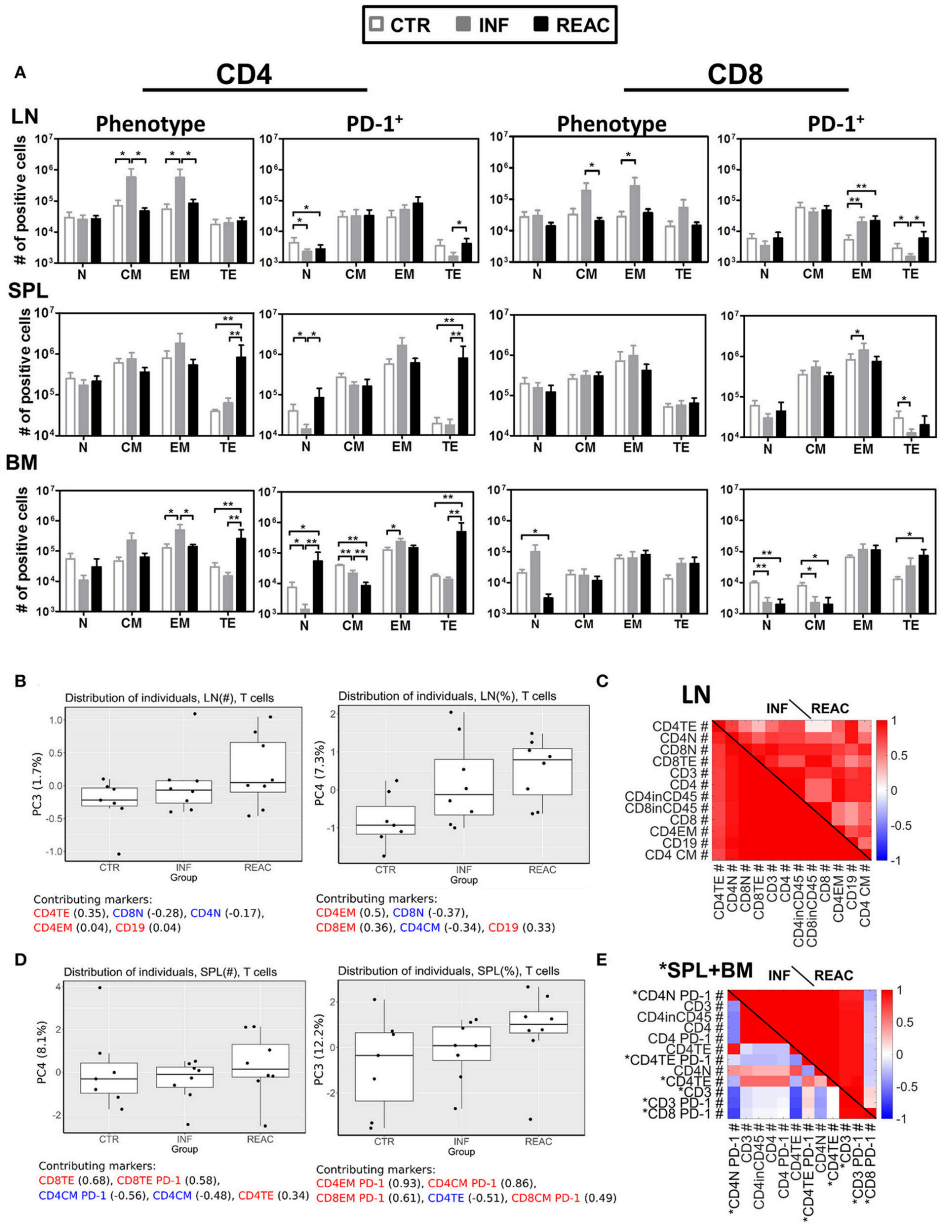
Differentiation of CD4^+^ and CD8^+^ T cell subsets after HCMV infections and reactivations and PCA analysis. **(A)** Total cell numbers (# of positive cells) are shown for CD4^+^ T cells (left) and CD8^+^ T cells (middle) for different subtypes (N, CM, EM, TE) and activation (PD-1^+^) (right) analyzed in CTR (white bars), INF (gray bars), or REAC (black bars) cohorts for LN, SPL, and BM. Reconstitutions included in the analysis are indicated (R1+R2: CTR *n* = 7, INF *n* = 8, REAC *n* = 8). Negative binomal-regression model was applied for statistical analysis (* < 0.05; ** < 0.01). **(B,C)** PCA analysis of T cell-associated markers with absolute numbers (#) was performed according to the materials and methods. Selected markers are sorted vertically (descending order) based on their maximum correlation with the first 3 principal components. Correlations between markers in **(B)** LN, and **(C)** combined measurements from SPL (blue) and BM (black) are shown for different groups of mice. **(B,C)** PCA performed with T cell-associated markers including absolute numbers (#) and frequencies (%) in LN was performed according to the materials and methods. **(B)** Distribution of the individual measurements in each group is shown for PC3 and PC4 as box-plots. The percentage of the data variation explained by each PC is given in the y-axis. The top 5 (ordered) markers contributing to the composition of each PC and their correlation with each PC are given. Positive and negative values of correlation coefficient are coded with red and blue texts, respectively. **(C)** Markers with highest maximum correlation with the first 3 PCs are selected and ordered vertically. Correlation between these markers is shown for INF and REAC groups as heat-maps. **(D,E)** The same analysis as in **(B,C)** was performed with T-cell associated absolute (#) and frequency (%) measurements in SPL **(D)** and using a combined data set of absolute numbers (#) of T-cell associated markers in SPL (marked with asterisk) and BM **(E)**.

Moving toward a more in-depth and complex analyses of the T cell signatures in tissues, we assessed the correlations between T cell immune phenotypic patterns with PD-1 expression (gating strategy provided in Figure S2). LN contained the highest numbers of CM and EM CD4^+^ and CD8^+^ T cells after HCMV infections (Figure 4A, Tables S6A–D, Figure S4A). The PD-1 expression pattern within the T cell subtypes in LN was between INF and REAC, but for T effector T cells of the INF cohort, PD-1 expression was lower. After HCMV reactivations, the numbers of TE CD4^+^ cells were significantly increased in SPL, BM, and SG, and this was associated with high numbers of CD4^+^ PD-1^+^ cells (Figure 4A, Tables S6A–D, Figures S3, S4A). Therefore, LN seemed to represent a distinct tissue regarding immunomodulation of T cells by HCMV. In general terms, these analyses emphasized the relevance of specific niches such as LN for central T cell memory homeostasis vs. SPL, BM, mLN, and LI for T cell activations toward PD-1^+^ activated effector T cells. Thus, the allocation of T cells with different maturation and activation status within different tissues showed a very complex pattern, likely defined by multifactorial variables. To dissect structural relationships (trends) between phenotypic markers and to uncover their changes according to the HCMV infection status, we employed PCA and correlation analyses. This approach evaluates any possible relationship between variables and its alteration (lost, formed or inversed) between the groups (CTR, INF, REAC). Due to a large set of measured quantities, we first employed PCA to identify the markers that best explain the data variations. This was achieved by considering all the groups simultaneously (INF, REAC, CTR). We tested all combinations of data subsets according to the organ and the type of measurements (frequencies, absolute numbers, B cell, or T cell associated markers), and only showed the data subsets that could enlighten the different groups.

Using the absolute (#) or frequency (%) of T cell associated markers in LN, we found two PCs that moderately separated REAC from INF and CTR groups. Terminal effector (#) and effector memory (%) CD4^+^ T cells in LN were important markers in these separating PCs (Figure 4B). The markers with absolute numbers gave a better separation of REAC group from INF and CTR compared to the frequencies, yet, the variation of the whole data was much smaller (compare the range of y-axis in Figure 4B). A separation of REAC from CTR and INF group could also be obtained using the SPL data (Figure 4D). Several important markers that contributed to these PCs contained PD-1. Then, the correlation analyses were performed between markers (absolute numbers #) within each group and the comparisons were depicted in the form of heat-maps (Figures 4C,E). The LN data of the INF and REAC groups were almost undistinguishable (Figure 4C), whereas the BM and SPL data showed clearly different interrelations of phenotypic markers between REAC vs. INF (Figure 4E). In sum, the PCA combined with the correlation analyses applied for immune modulation of T cells in SPL and BM distinguished reactivations from infections through a signature of memory and terminal effector T cells expressing the activation marker PD-1. This signature was less evident for LN data, where other markers such as CD19 were overrepresented for the REAC cohort.

### HCMV reactivations boost IgG^+^ memory B cells and plasma cells

**Figure 5:**
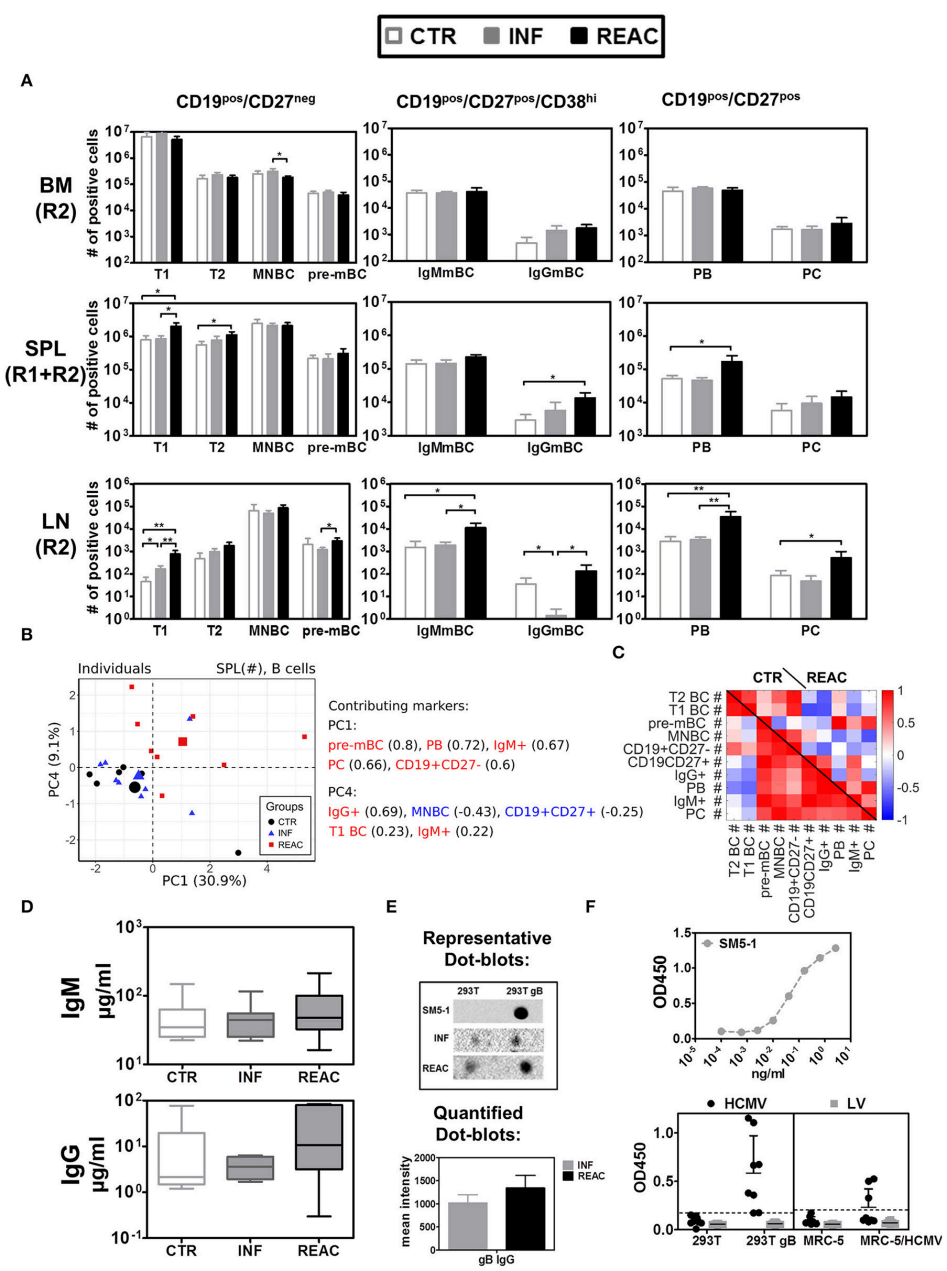
Differentiation of B cell subsets and class switch after HCMV infections and reactivations with HCMV specific IgG response. **(A)** Total cell counts (# of positive cells) are shown for B cells obtained from control (CTR, white bars), infected (INF, gray bars), or reactivated (REAC, black bars) cohorts in different tissues: LN (R2), SPL (R1+R2), and BM (R2). Error bars indicate standard error of the mean. Negative binomial-regression model was applied for statistical analysis (* < 0.05; ** < 0.01). **(B)** Similar PCA and correlation analyses to Figures 4B,C is shown for B cell associated markers with absolute numbers (#) in SPL. **(B,C)** PCA performed with B cell-associated markers consisting of absolute numbers (#) in SPL was performed according to the materials and methods. **(B)** Similar analysis as Figures 4B,**D** was performed and the individual measurements are shown according to their values in PC1 and PC4. **(C)** Similar correlation analysis as Figures 4C,E is presented in the form of heat-map for CTR and REAC groups. **(D)** Total human IgM and IgG (μg/ml) measured in plasma by ELISA for CTR (*n* = 7), INF (*n* = 7), and REAC (*n* = 7) cohorts. **(E)** Dot-blot assays. Plasma from HCMV infected mice was tested for reactivity against HCMV-gB based on cell extracts of 293T-w.t. and 293T-gB cells loaded as dot-blot on membranes which were exposed to plasma and secondary antibody and developed by chemiluminescence analyses. The mean intensity combining the results obtained for INF (*n* = 7) and REAC (*n* = 7) mice is shown. SM5-1 is human monoclonal antibody against gB used as a positive control. Plasma of CTR mice showed no signal. **(F)** ELISA assay performed with mouse plasma. ELISA plates were coated with cell extracts generated with 293T, 293T-gB, MRC-5, and MRC-5 infected with HCMV cells. Upper panel: Serial dilutions of the SM5-1 antibody was used as a reference in ELISA plates coated with 293TgB extracts. Lower panels: 1/10 diluted plasma samples obtained from humanized mice administered with MRC-5 cells infected with HCMV/GLuc (HCMV-REAC) or administered with MRC-5 cells infected with LV/GLuc (MRC-5-LV) (Exp. 2) were used. Reactivity of the antibodies were measured in the ELISA assay for absorbance and depicted as OD450. Each data point represents results obtained for individual mice of the HCMV-REAC (*n* = 4) or MRC-5 LV (*n* = 4) cohort. Error bars indicate standard deviation. Hatched line depicts the background signal of the assay.

Next, we evaluated the effects of HCMV infections and reactivations on the B cell development, maturation and class switch (Figure 5, Tables S7A,B). The B cell antigen CD19 assembles with the B cell receptor (BCR) and is continuously expressed along the different stages of early B cell development but it is lost upon maturation to plasma cells. On the other hand, CD27 is a later B cell marker, which is the ligand for CD70, and plays a key role in regulating B-cell activation and immunoglobulin synthesis. Since B cells initially develop in BM, this was the tissue initially analyzed. As expected, most of the B cells there were immature (CD19^pos^/CD27^neg^), and could be further sub-grouped in immature/ transitional type 1 (T1, CD24^hi^/CD38^hi^) (Figure S5B) cells, transitional type 2 (T2, CD24^int^/CD38^int^), mature naïve (MNBC, CD24^low^/CD38^low^), and pre-memory B cells (pre-mBC, CD24^pos^/CD38^neg^), whereas very few plasma blasts (PB, CD138^neg^), plasma cells (PC, CD138^pos^), and IgG^+^ cells could be detected (see gating strategy in Figure S2). Most of the detected CD19^pos^/ CD27^pos^ B cells in BM were IgM^+^ but IgG^+^ cells were also detectable. Thus, HCMV infections and reactivations did not alter much the pattern of B cell development in BM. In contrast, reactivations pushed the development and maturation of B cells in SPL, resulting in higher numbers of T1 (REAC vs. CTR *p* = 0.01, Table S7A), T2 (REAC vs. CTR *p* = 0.03, Table S7A), PB (REAC vs. CTR *p* = 0.03, Table S7B), and IgG^+^ B cells (REAC vs. CTR *p* = 0.01, Figure 5A, Table S7B, Figure S6A). The effects of HCMV reactivations obtained for SPL were in line with the results obtained for mLN and LI (Table S7B, Figures 5B). In contrast, LN showed a deviant pattern, as primary infections suppressed the development of IgG^+^ memory B cells (REAC vs. INF *p* < 0.01, Table S7B, Figure 5A) and reactivations pushed significantly the development of mature PB, PC, and IgG^+^ B cells (Figure 5A, Table S7B, Figure S6A). A PCA performed with B cell associated markers using absolute cell numbers (#) in SPL resulted in two separating PCs, which clearly distinguished REAC group from the other groups (Figure 5B). The phenotypic markers in PC1 (pre-MBC, PB, IgM^+^, PC, and CD19^+^CD27^+^) and PC4 (IgG^+^, T1 BC, and IgM^+^) were among the markers overrepresented in the REAC group. Therefore, the heat map based on the SPL data clearly showed different interrelations of phenotypic B cell markers in REAC vs. CTR (Figure 5C).

### Reactivations boost IgGs against HCMV-gB

We further evaluated whether the distinctive B cell-related immune phenotypic patterns associated with HCMV reactivations correlated with functional antibody generation. Levels of human IgM and IgG in plasma were both higher after HCMV reactivations (figure 5D, Table S8). The levels of human IgG (determined by least square mean estimation, LSM) were considerably higher after reactivations (27.89 μg/ml) relative to controls (15.37 μg/ml) or primary infections (6.44 μg/ml). In order to evaluate reactivity of human IgGs recovered from plasma against HCMV-gB, dot-blots using protein extracts obtained from wild type (w.t.) 293T cells and 293T cells expressing gB were used for immune detection (Figure 5E). No signs of IgG reactivity against gB could be found in plasma of control mice. Plasma obtained from mice after HCMV infections showed detectable reactivity, which was further enhanced with plasma from mice after HCMV reactivations. Further, an ELISA assay was developed in order to provide additional evidence by other methods that mice infected with HCMV and undergoing reactivations could mount IgG responses against HCMV (Figure 5F). ELISA plates were coated with protein lysates generated with 293Twt, 293TgB, MRC-5 cells, or MRC-5 cells infected with HCMV. As a reference for the assay we used a high affinity purified human monoclonal antibody recognizing gB (SM5-1). Plasma samples from mice obtained in experiment #2 (receiving either MRC-5/LV-GLuc or MRC-5/HCMV-GLuc) were diluted 10-fold and applied to the wells of the plates. The results showed clearly that plasma obtained from several mice undergoing HCMV reactivation reacted against proteins present in 293TgB and in MRC-5 cells infected with HCMV. Only background signals were obtained for plasma obtained from mice administered with MRC-5 cells infected with LV-GLuc. These results confirmed the data shown by Crawford et al using other ELISA methods (25) that humanized mice infected with HCMV can mount detectable IgG responses.

### LDA analyses allowed a decisive representation of T and B cell signatures

**Figure 6:**
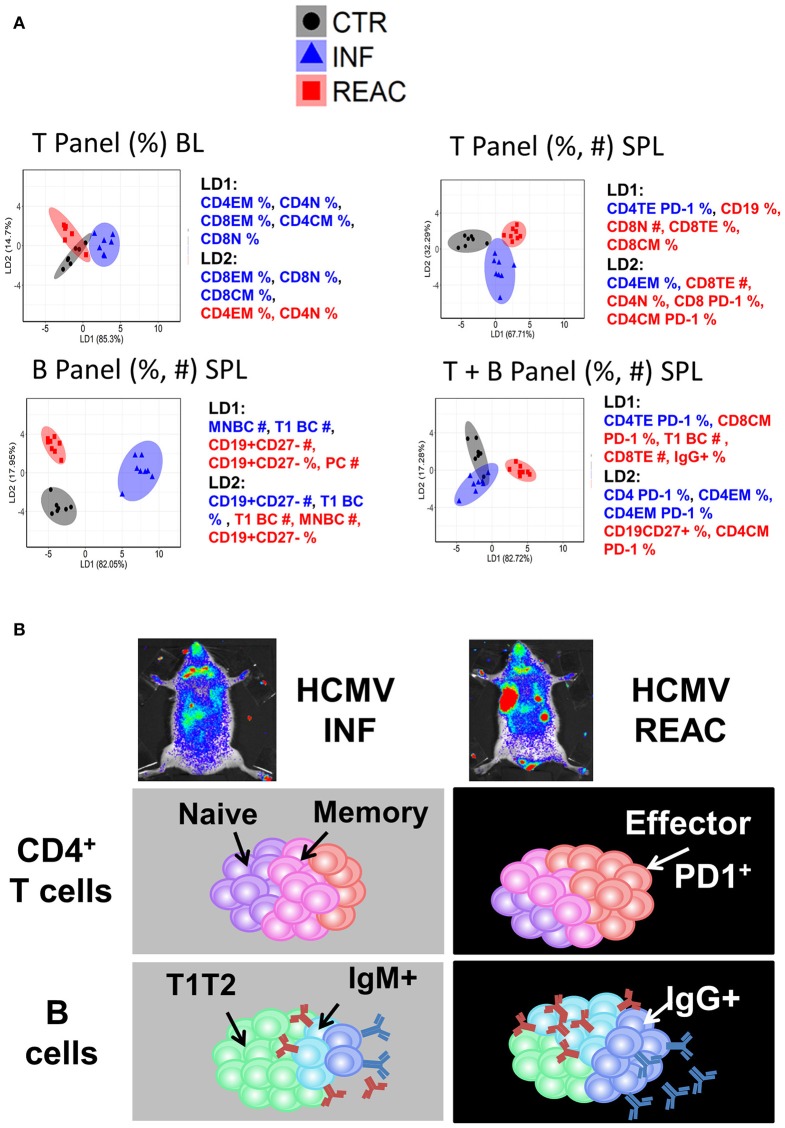
Distinctive immunologic signatures observed in humanized mice after HCMV infections and reactivations. **(A)** LDA performed with panels of immune phenotypic markers (frequencies % and absolute numbers #) used to characterize T and/or B cells measured in blood (BL) and spleen (SPL). The clusters show CTR in gray, INF in blue, and REAC in red. The axes represent the percentage separation achieved by each discriminant function (LD1, LD2) according to a “proportion of trace.” The top 5 markers with highest contributions (highest absolute coefficients) in the composition of discriminant functions are shown. Markers with negative and positive contributions are annotated in blue and red, respectively. **(B)** Schematic representation of the data explaining the model: Relative to the control group, infections promoted the development of memory CD4^+^ T cells whereas reactivations were strongly associated with expansion of effector CD4^+^ PD-1^+^ T cells and development of IgG^+^ mature B cells.

As another strategy to reduce the complexity of our data and find phenotypic markers that might be essential for defining HCMV infections vs. reactivations status, we employed LDA on our experimental data in similar fashion to PCA (see Methods). LDA performed with a subset of measurements (frequencies and/or absolute values) obtained from the T and B cell panels (individually or combined) resulted into distinct separations of CTR, INF, and REAC groups (Figure 6A). LDA analyses using only the frequencies of T lymphocyte types in blood identified clusters for each experimental arm, but with some overlap. When the frequencies and absolute numbers of T lymphocyte types in spleen were used in combination, the experimental arms could be better divided. Nevertheless, LDA performed with frequencies and absolute numbers of the B cell panel clearly showed the best distinction among the clustered groups. However, when the data obtained with B and T cell panels were combined, the clustering was less prominent, indicating that data obtained from T and B panels were not additive or synergistic. In summary, the LDA analyses advocated that using different subsets of immune phenotypic markers, and predominantly the B cell panel, mice with HCMV infections and reactivations could be distinctively distinguished from the control group.

## Discussion

Prior studies based on humanized models demonstrated HCMV infections and reactivations *in vivo* (20, 21, 25), but did not address their differences regarding tissue-dependent patterns of T and B cell development, differentiation and activation. Here, we described a practical, robust and reliable *in vivo* model discerning the effects of HCMV primary infections and reactivations on human T and B cell immune modulations and their functional responses against HCMV antigens. Our model takes a simplified approach for studying HCMV infections *in vivo* model using NRG female mice transplanted with CB purified CD34^+^ cells. When human T cell immune reconstitution stabilized at 17 weeks post-transplantation, cell carriers infected with HCMV/GLuc were administered and viral reactivations were promoted by hG-CSF administration. Expression of *Gaussia* luciferase as a reporter gene inserted downstream of the IE1 promoter into the HCMV genome allowed non-invasive bio-distribution analyses of viral dissemination and reactivations. The combination of optical imaging and PCR methodologies showed highest effects of viral reactivations in LI, in SG and, remarkably, LN. LN-like structures like the ones seen here were rarely documented by other groups developing humanized mouse models, because most of these studies were carried on using humanized mice too soon (10–12 weeks) after HSCT, when they are not detectable. The optical imaging approach provided a tool to directly point to the main sites of HCMV infections and reactivations to then study the systemic interplay between human T and B cell responses.

The information about the human T/B immune modulation in PBL was limited by the small volume of blood that could be gained from the mice allowing only analyses of relative cell frequencies, which were not very predictive. However, the availability of several different tissues engrafted with high numbers of human cells enabled a dynamic multidimensional examination of the effects of HCMV infections. In order to cope with the high dimensionality of multi-organ immune phenotypic profiles and complexity of their relation to the HCMV infection status, we employed PCA and LDA approaches to identify the sets of markers able to predict REAC groups. We employed different subsets of our experimental measurements to obtain possible combinations of predicting markers. Our analysis suggested that the structural differences of the HCMV infection and reactivation were sufficiently captured by the employed phenotypic markers, such that multiple subsets of markers were able to distinguish and immunologically explain the HCMV infection vs. reactivation status. Despite certain degree of overlap between the sample distributions in REAC and INF groups in our PCA analysis, the proposed markers would be plausible candidates for training more advanced machine learning techniques, such as artificial neural networks, to improve big-data clustering for elucidating future HCMV infections and reactivations models. However, LDA as a linear multivariate statistical method was a quite robust approach and sufficient to provide a clear separation between different groups using T and/or B cell markers in spleen as a single tissue to be analyzed. We tested the LDA results in the blood, which corresponds to a relevant source of clinical measurements in humans (Figure 6). T cell associated measurements provided a satisfactory separation of REAC from INF, whereas this separation was not obtained by PCA (data not shown). The performance of multiple data classifiers is generally evaluated and compared by using a validation data set, which cannot be performed in an unbiased manner in our study due to sample size limitation. Therefore, our LDA analyses are preliminary attempt to evaluate the feasibility of class separation and essential measurements using simple linear classifiers. Overall, the clear message coming out of our model with these combinations of analyses is that whereas primary HCMV infections promoted robust memory T cell responses, viral reactivations were associated with maturation/activation of effector T cells (Figure 6). This is supported by findings reported for the effects of HCMV in humans (12–14, 38, 39). Furthermore, reactivations resulted into enhanced numbers of PD-1^+^ CD4^+^ TE T cells in SPL and BM. This also corroborates clinical observations, as for patients after HSCTs that were non-viremic, with prolonged viremia or with CMV disease, significant upregulation of PD-1 on T cells obtained from blood was shown of the latest group (40). In our model, reactivations seemed to be also associated with reduced total numbers of effector CD8^+^ T cells, possibly because they were chronically activated resulting into exhaustion and death. On the other hand, infections stimulated B cell development, maturation and class switch in several tissues. Nonetheless, these effects were notably amplified by viral reactivations, probably as a result of an improved CD4^+^ T cell maturation (Figure 6). Combining the T and B cell responses derived from our model, we can extrapolate to other clinical observations: Patients who underwent a single reactivation generated significantly higher amounts of CD4^+^ IFN-γ^+^ cells and humoral dominated immune response (functional against gB), than did patients with multiple reactivation episodes (15); after primary infections, HCMV-neutralizing antibody levels were high in transplanted patients from 6 to 12 months after transplant (16) and circulating memory Tfh defined as CXCR5^+^ CD4^+^ cells were identified concomitantly to both neutralizing and IgG antibodies to HCMV glycoproteins (17, 41).

One important new finding observed in this model was that HCMV infections promoted also thymic development of CD4^+^ T cells and this was not lessened after viral reactivations. The development of functional and HLA-restricted T cells in humanized mice is currently perceived as enigmatic, since the stroma cells that should provide antigenic stimulus, and support for the TCR rearrangements during thymopoiesis in the mouse thymus are mouse derived. Thus, they express mouse (not human) major histocompatibility complexes (MHCs) and the antigen presentation should be restricted to the mouse MHCs. Yet, in previous work using NRG mice humanized with CB-CD34^+^ cells from HLA-A:02.01 donors and immunized with matched induced DCs expressing pp65 (iDCpp65), we obtained T cells from spleen or lymph nodes, expanded them *in vitro* for a week with iDC or iDCpp65 and then preformed the ELISpot using K562 cells expressing HLA-A * 02:01 or co-expressing pp65 and HLA-A * 02:01 (26, 27). We could conclusively demonstrate HLA-A * 02:01-restricted responses with these assays. The underlying mechanism remains as a conundrum. In immunocompetent mice, the thymus supports T-cell development, but also contains non-T-cell lineages such as dendritic cells, macrophages, and granulocytes that are necessary for T-cell repertoire selection and apoptotic thymocyte clearance. Thus, we hypothesize that human DCs or B considered as good antigen presenting cells (APCs) highly expressing class I and class II HLAs cells and trafficking or homing in the thymus and could also support HLA-restricted T cell development. The observed stimulation of thymic development by HCMV observed here in humanized mice may be linked to the presentation of HCMV antigens by human APCs to early T cell progenitors. This may clarify the clinical paradox whether HCMV reactivations and positive HCMV serology are beneficial (42) or detrimental (43, 44) to stem cell recipients regarding the “graft vs. leukemia” (GVL) effect, as this may be time dependent. If HCMV reactivations occurs too early (5–6 weeks after HSCT), CD4^+^ T cells development in thymus may not be effective yet, whereas if HCMV reactivation occurs later (such as in our humanized mouse model), it might actually improve the overall development of CD4^+^ T cells to assist in GVL effects. Large retrospective clinical studies concluded that HCMV reactivations occurring early after mobilized blood- or CB-HSCT for patients with hematological malignancies were associated with lower overall survival and higher non-relapse mortality (43, 44). Paradoxically, others reported that HCMV reactivations correlated with substantially improved reduction of relapse incidence in patients with acute myeloid leukemia (AML) after transplantation (42). This phenomenon was explained either as a direct “virus-vs.-leukemia” infection effect or as an indirect immune stimulatory effect of HCMV on the innate and adaptive immune system responses, which in turn could improve GVL effects. But this conundrum is unresolved, as the descriptive nature of these studies limited so far the identification of mechanisms underlying the effects of HCMV infections or reactivations in the immune system and *vice-versa* (45). Thus, a HCMV mouse model combined with challenge of human leukemia could provide a valuable experimental platform to examine the highly disputed clinical enigma whether mechanisms of HCMV latency and reactivations can combat or predispose human leukemia development.

The data obtained in our humanized HCMV model also supports early findings obtained by Jonjic et al. with mice infected with murine CMV (MCMV), demonstrating that B cells and probably antibodies restricted dissemination of reactivated virus thus limiting recurrent infection (46). Using the MCMV one of the co-authors in our studies recently showed that a number of neutralizing anti-gB monoclonal antibodies could bind to similar antigenic structures on MCMV-gB that are represented in HCMV-gB, reducing viral burden in immunodeficient RAG^−/−^ hosts to limit an ongoing infection (47). Our findings in humanized mice showed that B cell and humoral responses were not inhibited by viral reactivations, further endorsing the development of new vaccines boosting humoral responses, as currently being evaluated in clinical trials (48) or, alternatively, with adoptive administration of B cells reactive against HCMV (49). On the other hand, we observed that HCMV reactivations resulted into increased numbers of terminally differentiated and activated CD8^+^PD-1^+^ cytotoxic T lymphocytes (CTLs). This is important, as HCMV-reactive adoptive memory CTLs used for HCMV immune cell therapy (50–52) could potentially become chronically activated and dysfunctional after infusion and migration to lymphatic tissues harboring HCMV reactivation. Similarly as previously validated for EBV pathologies in humanized mice (53), this novel HCMV infection model and advanced multivariate analyses systems can be used as proof-of-principle to evaluate innovative immune therapies such as check-point inhibitors blocking PDL-1 and CTLA-4.n the future, it will be interesting to evaluate the effects of HCMV infections in immune deficient mouse strains expressing human MHCs or multiple human cytokines (54).

Undoubtedly, there are several limitations associated with humanized mouse models that remain to be addressed [recently reviewed in (54)]. Structural limitations such as the mouse stroma present in the thymus discussed above or the poorly organized lymph nodes that are eventually repopulated with human cells several weeks (>14) after HSCT are difficult to be solved.

Nevertheless, a continued need exists to identify species-specific differences between human and mouse cytokines and other factors that are absent in mice and needed for human immune cell differentiation and function. Thus, human cytokines are being provided as administered recombinant factors, as transgenic or knock-in approaches into mice. Providing human IL-7 alone stimulated human T cell development in humanized BALB/c-Rag2^−/−^ mice, but did not support peripheral T cell maintenance (55). Later, it was shown that naïve and memory phenotype T cell subsets could be significantly increased by exogenous administration of a potent human IL-15R agonist into humanized (54) BALB/c-Rag2^−/−^γc^−/−^ mice and this was associated with improved humoral responses (56). More recently, it was shown that provision of human IL-6 to knockin mice (Rag2^−/−^ γc^−/−^IL6^h/h^ or with SIRPα knock-in Rag2^−/−^ γc^−/−^ SIRPα^h/m^ IL6^h/h^) humanized with fetal liver CD34^+^ cells injected into the liver of newborn mice greatly enhanced human thymopoiesis, periphery T-cell development, and also significantly increased class switched memory B cells and serum IgG upon immunizations with OVA (57). As these new approaches improve the human adaptive responses in humanized mice, it would be interesting to evaluate in these models the T and B cell responses produced by HCMV infections and reactivations.

In addition, future humanized mouse models can be further complemented with CB-matched human endothelial or mesenchymal cells to promote and maintain HCMV lytic cycles. The development of drugs used for prevention and treatment of HCMV infections after allo-HSCT has recently been broadened from ganciclovir and foscarnet (58) to letermovir (57) and maribavir (59). Nevertheless, these drugs have considerable side effects (including immunological effects) in certain patient populations and do not protect patients against later onset HCMV infections. Passive immunization by administration of HCMV immunoglobulin (CMVIG) preparations aims to restore normal concentrations of HCMV-specific immunoglobulin in the immunocompromised patient but data as monotherapy is still limited and its mode of action in the immune system is not yet fully understood (60, 61). Several HCMV-specific monoclonal antibodies are in the research pipeline (62, 63) or entering clinical trials for assessing efficacy, pharmacokinetic and anti-drug antibody evaluations (64). Incremental advances in the generation and selection of HCMV-reactive adoptive T cells has convincingly improved the survival of high-risk patients (50–52, 65, 66). Despite these pharmacologic and immune cell therapies, HCMV disease can still cause considerable morbidity and mortality (3–5). Availability of reproducible and predictive humanized mouse models of HCMV infections and reactivations such as the one shown here are therefore required to demonstrate proof-of-concept, efficacy and safety of single agent or combination therapies.

In summary, this novel humanized mouse model recapitulated and differentiated HCMV primary infections and reactivations after HSCT and showed multifaceted tissue-dependent patterns of immune modulatory effects. Convincingly, it showed that HCMV infections and reactivations resulted functional T and B cell responses against HCMV antigens which could be split into distinct signatures: infections promoted differentiation and activation of memory T cells whereas reactivations promoted differentiation and maturation of B cells. Our data suggest that PD-1 upregulation is predictive of HCMV reactivations and can become a practical and useful biomarker. In summary, this novel experimental model allied with multivariate analyses reflects the complexity and diversity of clinical outcomes after HCMV infections and reactivations and can be explored for testing efficacy of novel antiviral therapies and vaccines.

## Author contributions

ST conducted experiments, analyzed data, and wrote the first manuscript draft. SK and MM-H performed the PCA and LDA analyses, interpreted the data, wrote, and edited the manuscript. VV, HO, SD, LG, and AS assisted in preparation and analyses of humanized mice. CS provided the HCMV/GLuc strain. DS performed the pathology analyses. SL, PR, and CG participated in the optical imaging analyses. CF performed the human cytokine array analyses. CvK assisted in the procurement and collection of HSC for the studies. LS performed the statistical analyses. SG and AM-B provided the panels for B cell analyses. AG. and MS provided clinical input. MMa and MMe provided technical assistance for analyses of HCMV. RS planed the project, designed experiments, obtained funding and regulatory approvals, enrolled collaborators, interpreted the data, wrote, and edited the manuscript.

### Conflict of interest statement

RS received honoraria as a lecturer in conferences from The Jackson Laboratory, which commercially distributes the NRG mice. The remaining authors declare that the research was conducted in the absence of any commercial or financial relationships that could be construed as a potential conflict of interest. The handling Editor declared a past co-authorship with one of the authors MMe.
