# Supplementary material for: Signatures of T and B Cell Development, Functional Responses and PD-1 Upregulation After HCMV Latent Infections and Reactivations in Nod.Rag.Gamma Mice Humanized With Cord Blood CD34+ Cells
Source: Front Immunol. 2018 Nov 22;9:2734. doi: 10.3389/fimmu.2018.02734 (PMC6262073; doi:10.3389/fimmu.2018.02734)
Supplement: Supplementary file 1 [file Data_Sheet_1.docx]

**Signatures of T and B cell development, functional responses and PD-1 upregulation after HCMV latent infections and reactivations in Nod.Rag.Gamma mice humanized with cord blood CD34^+^ cells**

**Running Title: HCMV immune modulates humanized mice**

Sebastian J. Theobald^1,2,3^, Sahamoddin Khailaie^4,5^, Michael Meyer-Hermann^5^, Valery Volk^1,2^, Henning Olbrich^1,2,3^, Simon Danisch^1,2,3^, Laura Gerasch^1,2^, Andreas Schneider^1,2^, Christian Sinzger^6^, Dirk Schaudien^7^, Stefan Lienenklaus^8^, Peggy Riese^9^, Carlos A. Guzman^9^, Constanca Figueiredo^10^, Constantin von Kaisenberg^11^, Loukia M. Spineli^12^, Stephanie Glaesener^1^, Almut Meyer-Bahlburg^13^, Arnold Ganser^1^, Michael Schmitt^14^, Michael Mach^15^, Martin Messerle^3,16^, and Renata Stripecke^1,2,3*^

^1^ Clinic of Hematology, Hemostasis, Oncology and Stem Cell Transplantation, Hannover Medical School, Hannover, Germany.

^2^ Laboratory of Regenerative Immune Therapies Applied, Excellence Cluster REBIRTH, Hannover Medical School, Hannover, Germany.

^3^ German Center for Infection Research (DZIF), Partner Site Hannover-Braunschweig, Germany.

^4^ Department of Systems Immunology and Braunschweig Integrated Centre of Systems Biology (BRICS), Helmholtz Centre for Infection Research, Braunschweig, Germany

^5^ Institute for Biochemistry, Biotechnology and Bioinformatics, Technical University Braunschweig, Braunschweig, Germany

^6^ Institute of Virology, University of Ulm, Germany.

^7^ Fraunhofer Institute for Toxicology and Experimental Medicine (ITEM), Hannover, Germany

^8^Institute for Laboratory Animal Science, Hannover Medical School, Hannover, Germany.

^9^Department of Vaccinology and Applied Microbiology, Helmholtz Centre for Infection Research Braunschweig, Germany.

^10^Department of Transfusion Medicine, Hannover Medical School, Hannover, Germany.

^11^Clinic of Gynecology and Obstetrics, Hannover Medical School, Hannover, Germany.

^12^Institute for Biostatistics, Hannover Medical School, Hannover, Germany.

^13^Department of Pediatrics, University medicine Greifswald, Greifswald, Germany

^14^Department of Hematology, Oncology and Rheumatology, GMP Core facility, Heidelberg University Hospital, Heidelberg, Germany.

^15^ Institute of Virology, University Erlangen-Nürnberg, Erlangen, Germany

^16^Institute of Virology, Hannover Medical School, Hannover, Germany

*** Correspondence:** Corresponding Author stripecke.renata@mh-hannover.de

Keywords: HCMV, reactivation, humanized mice, T cell maturation, B cell class switch, optical imaging analyses, principal component analyses, linear discriminant analyses.

## Supplementary Figures


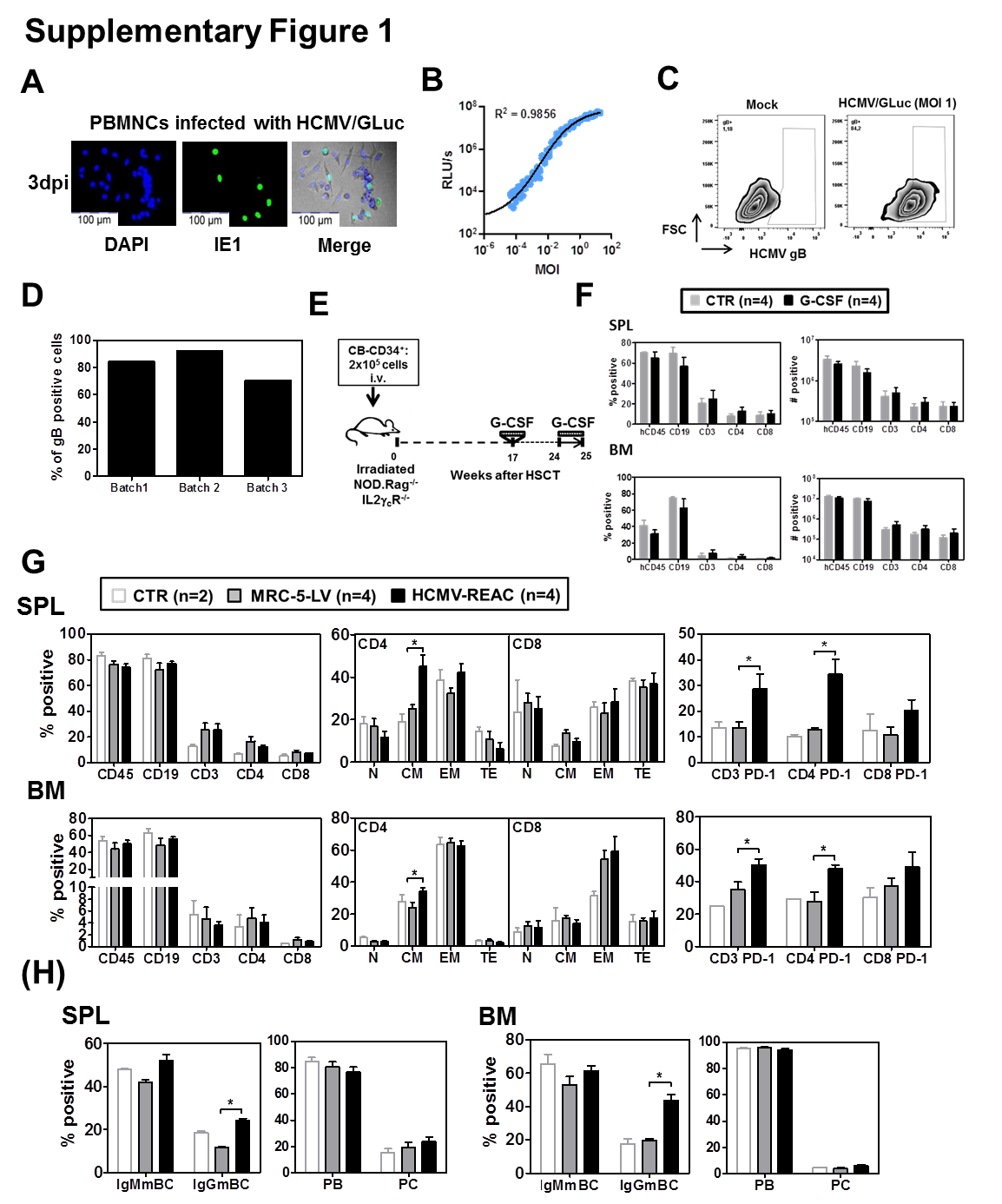


**Supplementary Figure 1.** (A) PBMNCs mock or infected with HCMV-GLuc (MOI=0.5) were stained for immune detection of HCMV/IE1 (green) and nuclei by DAPI (blue) and analyzed by immune fluorescence microscopy. Scale bars are indicated and 20x objective was used (B) MRC-5 cells were infected with HCMV-GLuc at different MOIs and secreted luciferase in cell supernatants produced bioluminescence signals analyzed by luminometry corresponding to Relative Light Units (RLU). The regression coefficient is indicated in the graph. (C) Representative example and (D) analyses of three batches of MRC-5 cells infected with HCMV-GLuc at MOI 1, cryopreserved, thawed and analyzed by flow cytometry for gB expression. (E) Experimental scheme of hG-CSF test in humanized NRG mice. (F) NRG mice were transplanted with 4 different cord blood CD34^+^ cells (CTR, n=4) and for a second group hG-CSF was administered into using the same experimental set up but without HCMV infection (hG-CSF, n=4). At week 25 after HSCT, spleen an bone marrow were analyzed by flow cytometry for quantification of total numbers of huCD45^+^, CD19^+^ within huCD45^+^, CD3^+^ within huCD45^+^, CD4^+^ within huCD45^+^ and CD8^+^ within huCD45^+^. Two way ANOVA with bonferri post-test was applied for statistical analysis. No significant values were observed. (G) Percentage of positive cells are shown for SPL and BM for the experiment comparing the effect of MRC-5-LV vs. HCMV-REAC for T cell phenotypes and for (H) B cells.


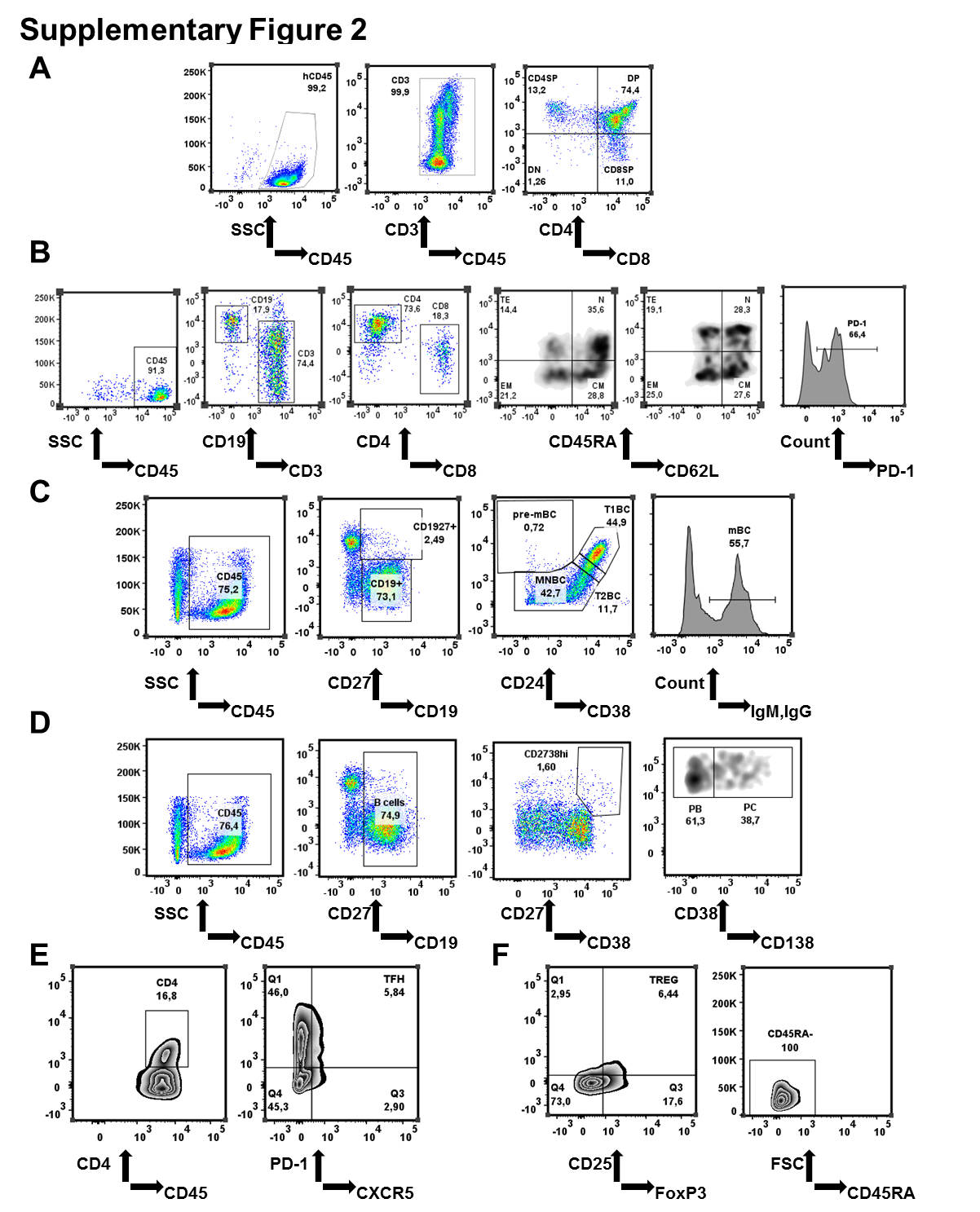


**Supplementary Figure 2.** (A) Representative flow cytometry gating example for the analyses thymus. (B) Representative flow cytometry gating example for the analyses T cells (CD3, CD4 and CD8), T cell subtypes (naïve, central memory, effector memory and terminal effectors) and PD-1^+^ expression on T cells. (C) Flow cytometry gating strategy of B cells and memory B cells. (D) Gating example for PC and PB in B cell population. (E) Representative flow cytometry gating example for the analysis of follicular T helper cells and (F) regulatory T cells.


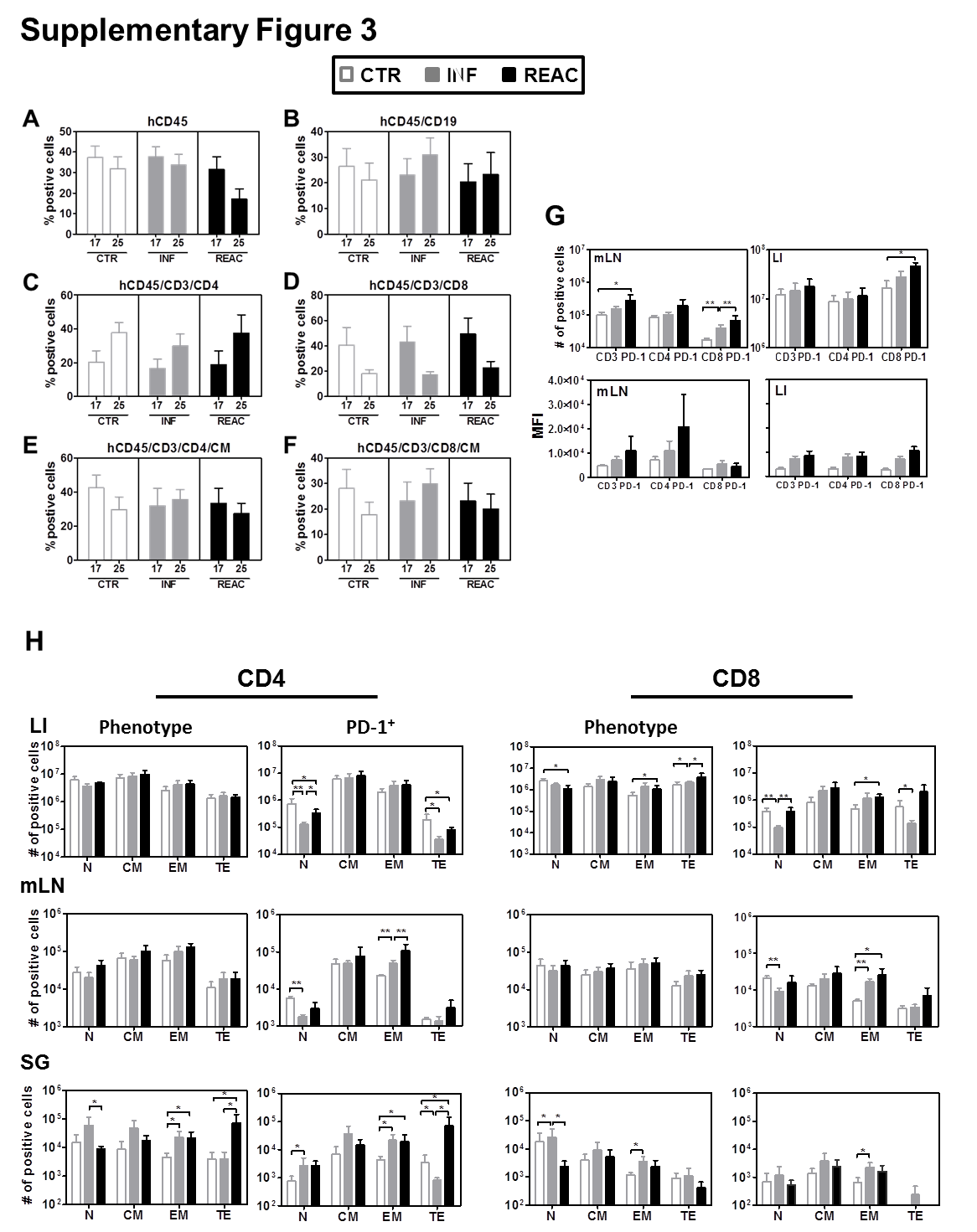


**Supplementary Figure 3.** Analyses if T cells in blood (A-F) determined at week 17 and 25 post transplation. % positive cells for hCD45 (A), CD19 (B), CD4 (C), CD8 (D), CD4CM (E) and CD8CM (F) are depicted. (G) Total cell numbers (# of positive cells) (top panel) and mean fluorescent intensity (MFI, bottom panel) for PD-1 expression on CD3^+^, CD4^+^ and CD8^+^ T cells in mLN (R2) and LI (R2). Negative binomal-regression model was applied for statistical analysis (*<0.05; **<0.01). (H) Total cell numbers (# of positive cells) for CD4 (left) and CD8 (right) T cell subtypes (naïve, central memory, effector memory and terminal effectors and PD-1 expression on CD4 and CD8 subtypes. Data is depicted for control (CTR, white bars), infected (INF, grey bars) or mice after reactivations (REAC, black bars) cohorts in different tissues: LI (R2), mLN (R1-R2) and SG (R2). Error bars indicate standard error of the mean. Non-bionmial-regression model was applied for statistical analysis (*<0.05; **<0.01).


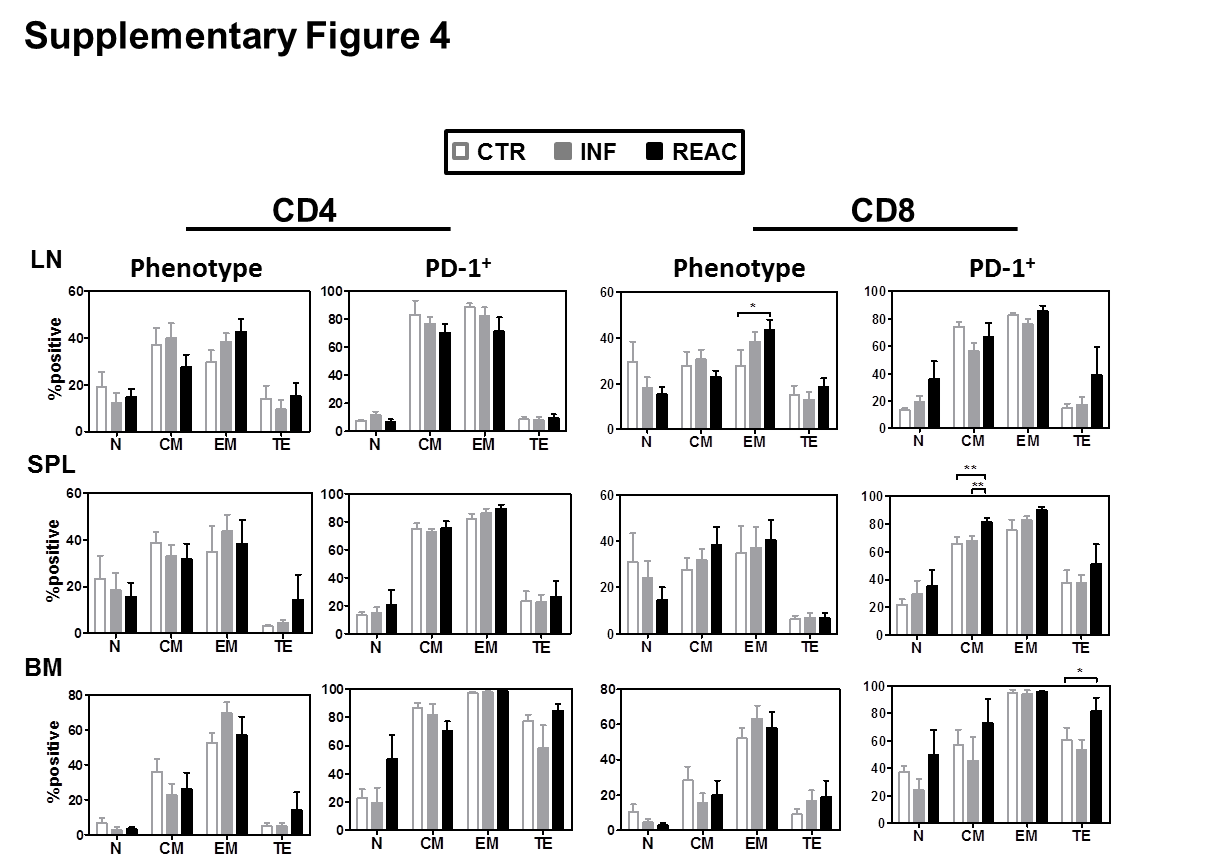


**Supplementary Figure 4.** (A) Percentage of positive cells (% positive) are shown for CD4^+^ T cells (left) and CD8^+^ T cells (middle) for different subtypes (N, CM, EM, TE) and activation (PD-1^+^) (right) analyzed in CTR (white bars), INF (grey bars) or REAC (black bars) cohorts for LN, SPL and BM.


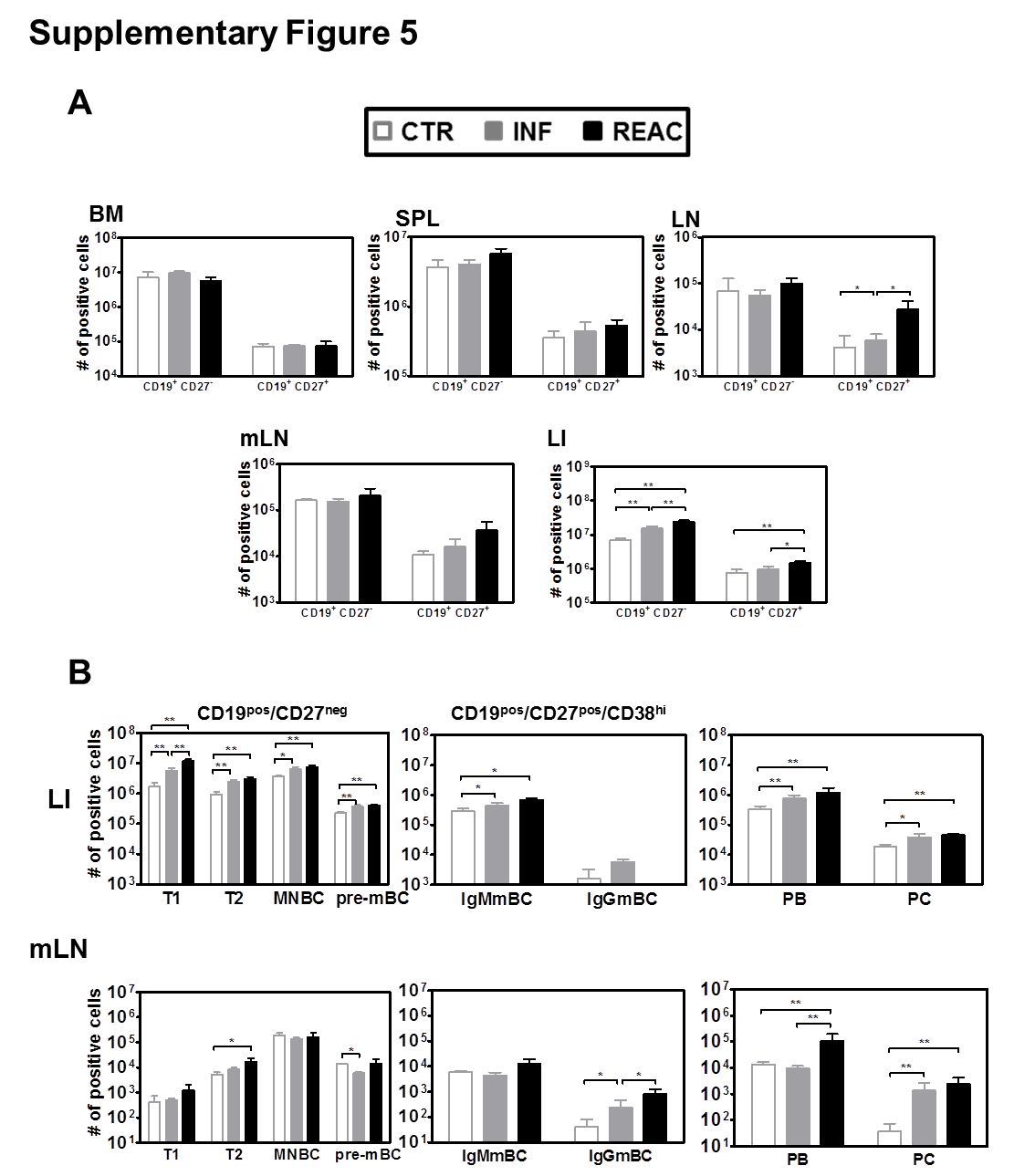


**Supplementary Figure 5.** (A) Total cell numbers (# of positive cells) (CD19^+^/CD27^-^ and CD19^+^/CD27^+^) are shown for cells obtained from control (CTR, white bars), infected (INF, grey bars) or mice after reactivations (REAC, black bars) cohorts in different tissues: Bone marrow (BM, R2), Spleen (SPL, R1+R2), Lymph nodes (LN, R2), Mesenteric lymph node (mLN, R2), Salivary glands (SG, R2) and Liver (LI, R2). (B) B cell analysis for LI (R2) and mLN (R2) depicted as # of positive cells. Error bars indicate standard error of the mean. Non-bionmial-regression model was applied for statistical analysis (*<0.05; **<0.01).


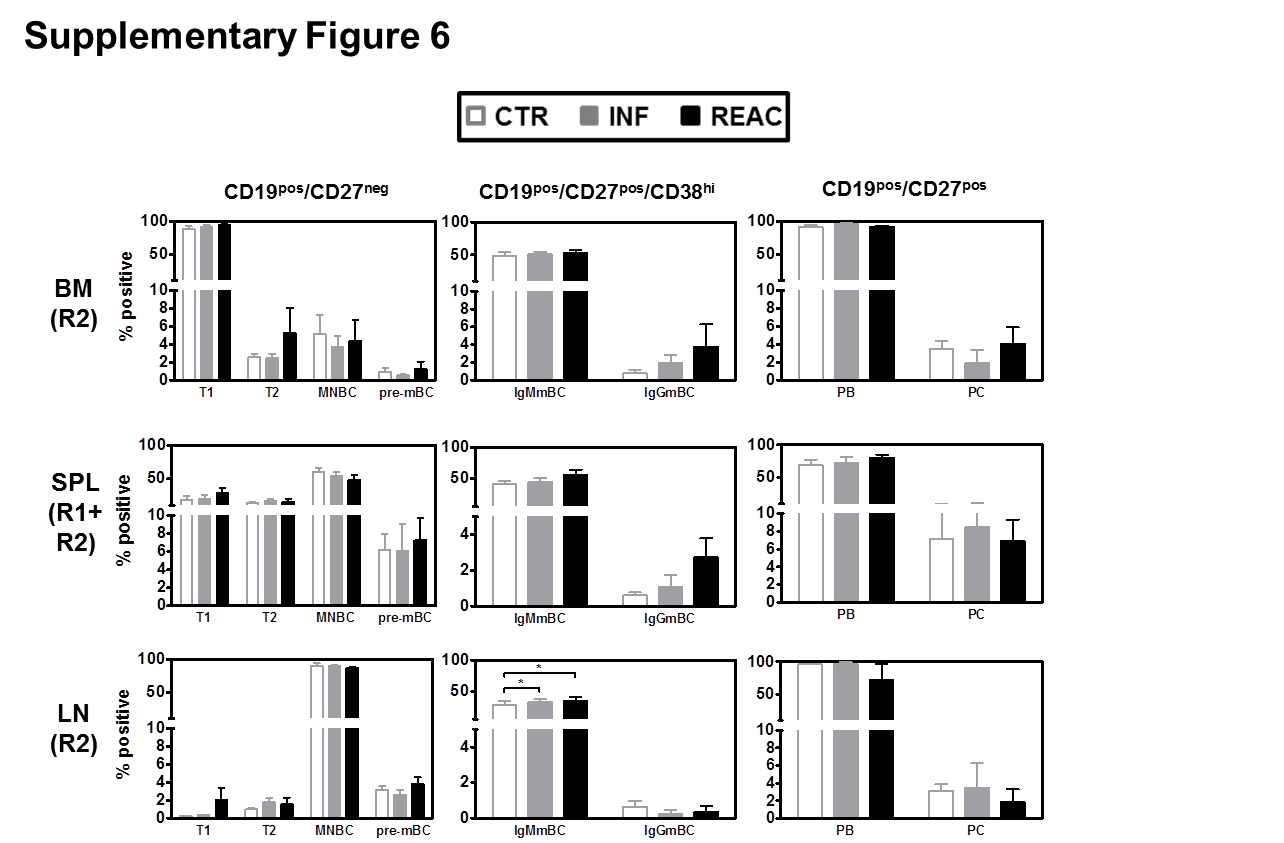


**Supplementary Figure 6.** (A) Frequencies of cell counts (% positive) are shown for B cells obtained from control (CTR, white bars), infected (INF, grey bars) or reactivated (REAC, black bars) cohorts in different tissues: LN (R2), SPL (R1+R2) and BM (R2). Error bars indicate standard error of the mean.

**Supplementary Table 1: Antibodies used for FACS analyses**

| Antigen | Fluorochrome | Company | Dilution | Catalogue # |
| --- | --- | --- | --- | --- |
| CD11c | APC | BD | 1:50 | 559877 |
| CD14 | FITC | Miltenyi | 1:25 | 130-080-701 |
| CD169 | PE | BD | 1:25 | 565248 |
| CD24 | FITC | BD | 1:100 | 555427 |
| CD27 | APC-Cy7 | Biolegend | 1:100 | 302816 |
| CD279 (PD-1) | PE | Biolegend | 1:100 | 329906 |
| CD3 | BV510 | Biolegend | 1:100 | 300448 |
| CD4 | PerCP | Biolegend | 1:250 | 317432 |
| CD45 | Pacific Blue | Biolegend | 1:100 | 304022 |
| CD45 | AL700 | Biolegend | 1:100 | 304024 |
| CD45RA | FITC | Beckman Coulter | 1:100 | A07786 |
| CD62L | PE-Cy5 | Beckman Coulter | 1:100 | IM26554 |
| CD8a | PE-Cy7 | Biolegend | 1:100 | 300914 |
| IgA | FITC | Thermo | 1:100 | H14001 |
| IgA | PE | Miltenyi | 1:100 | 130-093-128 |
| IgM | Pacific Blue | Biolegend | 1:100 | 314514 |
| IgG | PE-Cy7 | BD | 1:50 | 561298 |
| CD38 | APC | Biolegend | 1:50 | 303510 |
| CD138 | PE | Miltenyi | 1:100 | 130-101-168 |
| CD19 | AL700 | Biolegend | 1:250 | 302226 |
| CD19 | PerCP-Cy5.5 | Biolegend | 1:100 | 302230 |
| FoxP3 | PE | BD | 1:50 | 560046 |
| CD25 | APC | Biolegend | 1:100 | 101909 |
| CD 195 (CXCR5) | PE-Cy7 | BD | 1:25 | 313706 |
| CD34 | APC | Biolegend | 1:50 | 343606 |
| Anti-human IgG | HRP | Roth | 1:500 | 47531 |
| Anti-mouse IgG | AL647 | Biolegend | 1:250 | 405322 |
| Anti-mouse IgG | AL488 | Biolegend | 1:250 | 405319 |
| p27-287 (gB) | - | M. Mach | 1:20 | - |
| p63-27 (IE1) | - | M. Mach | 1:100 | - |
| HN | - | Merck Millipore | 1:100 | MAB4383 |
| CD3 | - | Dako | 1:50 | M7254 |
| CD79a | - | Dako | 1:25 | M7051 |
| Anti-mouse IgG | Biotin-SP | Jackson | 1:200 | 115-065-207 |

**Supplementary Table 2: Descriptive statistics regarding analyses of human cytokines detected in plasma.** LSM: least squares means estimation; MD: difference of the means (between CTR and INF; CTR and REAC; INF and REAC).

| **Cytokines** | **CTR** | | **INF** | **CTR** | | **REAC** | **INF** | **REAC** |
| --- | --- | --- | --- | --- | --- | --- | --- | --- |
| **INF γ** |  | | | | | | | |
| LSM | 395.93 | | 78.98 | 395.93 | | 385.07 | 78.98 | 385.07 |
| MD | 316.95 | | | 10.86 | | | -306.09 | |
| p-value | 0.07 | | | 0.95 | | | 0.11 | |
|  |  | | |  | | |  | |
| **GM-CSF** |  | | | | | | | |
| LSM | 309.85 | | 126.83 | 309.85 | | 291.83 | 126.83 | 291.83 |
| MD | 183.02 | | | 18.02 | | | -165.00 | |
| p-value | 0.26 | | | 0.91 | | | 0.33 | |
|  |  | | |  | | |  | |
| **IL-12** |  | | | | | | | |
| LSM | 19.91 | 5.92 | | 19.91 | 20.62 | | 5.92 | 20.62 |
| MD | 13.99 | | | -0.71 | | | -14.70 | |
| p-value | 0.36 | | | 0.96 | | | 0.35 | |
|  |  | | |  | | |  | |
| **TNF-α** |  | | | | | | | |
| LSM | 138.52 | 64.52 | | 138.52 | 109.72 | | 64.52 | 109.72 |
| MD | 74.00 | | | 28.80 | | | -45.20 | |
| p-value | 0.30 | | | 0.70 | | | 0.56 | |

**Supplementary Table 3: Descriptive statistics regarding analyses of thymus from NRG mice.** Note: LSM: least squares means estimation; RR: relative rate (between REAC and CTR; INF and CTR; REAC and INF).

| **Marker** | **REAC** | **CTR** | **INF** | **CTR** | **REAC** | **INF** |
| --- | --- | --- | --- | --- | --- | --- |
| **CD45** |  | | | | | |
| LSM | 3519708 | 1760880 | 2736805 | 1760880 | 3519708 | 2736805 |
| RR | 2.00 | | 1.55 | | 1.29 | |
| p-value | 0.15 | | 0.29 | | 0.49 | |
|  |  | |  | |  | |
| **CD3** |  | | | | | |
| LSM | 3406128 | 1693812 | 2629752 | 1693812 | 3406128 | 2629752 |
| RR | 2.01 | | 1.55 | | 1.29 | |
| p-value | 0.17 | | 0.33 | | 0.51 | |
|  |  | |  | |  | |
| **CD4SP** |  | | | | | |
| LSM | 512574 | 175463 | 569959 | 175463 | 512574 | 569959 |
| RR | 2.92 | | 3.25 | | 0.90 | |
| p-value | ***0.01*** | | ***0.01*** | | 0.85 | |
|  |  | |  | |  | |
| **CD8SP** |  | | | | | |
| LSM | 302256 | 268028 | 294193 | 268028 | 302256 | 294193 |
| RR | 1.13 | | 1.10 | | 1.03 | |
| p-value | 0.79 | | 0.82 | | 0.94 | |
|  |  | |  | |  | |
| **DN** |  | | | | | |
| LSM | 216249 | 81521 | 174087 | 81521 | 216249 | 174087 |
| RR | 2.65 | | 2.13 | | 1.24 | |
| p-value | ***0.02*** | | 0.12 | | 0.64 | |
|  |  | |  | |  | |
| **DP** |  | | | | | |
| LSM | 2444252 | 1223640 | 1671498 | 1223640 | 2444252 | 1671498 |
| RR | 2.00 | | 1.37 | | 1.46 | |
| p-value | 0.22 | | 0.56 | | 0.41 | |

**Supplementary Table 4: Descriptive statistics regarding analyses various organs for immune phenotype of NRG mice.** Note: LSM: least squares means estimation; RR: relative rate (between REAC and CTR; INF and CTR; REAC and INF).

|  | **SPL** | | | | | | | **LN** | | | | | |
| --- | --- | --- | --- | --- | --- | --- | --- | --- | --- | --- | --- | --- | --- |
|  | **REAC** | **CTR** | **INF** | **CTR** | **REAC** | **INF** | | **REAC** | **CTR** | **INF** | **CTR** | **REAC** | **INF** |
| **CD45** |  | | | | | | |  | | | | | |
| **LSM** | 9200413 | 8140280 | 9855930 | 8140280 | 9200413 | 9855930 | | 405749 | 398919 | 2170241 | 398919 | 405749 | 2170241 |
| **RR** | 1.13 | | 1.21 | | 0.93 | | | 1.02 | | 5.44 | | 0.19 | |
| **p-value** | 0.55 | | 0.49 | | 0.79 | | | 0.96 | | ***0.04*** | | ***0.03*** | |
|  |  | | | | | | |  | | | | | |
| **CD3** |  | |  | |  | | |  | |  | |  | |
| **LSM** | 4001957 | 4026212 | 5458888 | 4026212 | 4001957 | | 5458888 | 288417 | 300449 | 1874600 | 300449 | 288417 | 1874600 |
| **RR** | 0.99 | | 1.36 | | 0.73 | | | 0.96 | | 6.24 | | 0.15 | |
| **p-value** | 0.98 | | 0.45 | | 0.48 | | | 0.91 | | ***0.03*** | | ***0.02*** | |
|  |  | | | | | | |  | | | | | |
| **CD4** |  | |  | |  | | |  | |  | |  | |
| **LSM** | 1964468 | 1441409 | 2284006 | 1441409 | 1964468 | | 2284006 | 181597 | 172488 | 1126721 | 172488 | 181597 | 1126721 |
| **RR** | 1.36 | | 1.58 | | 0.86 | | | 1.05 | | 6.53 | | 0.16 | |
| **p-value** | 0.41 | | 0.27 | | 0.78 | | | 0.90 | | ***0.03*** | | ***0.03*** | |
|  |  | | | | | | |  | | | | | |
| **CD8** |  | |  | |  | | |  | |  | |  | |
| **LSM** | 1434050 | 2139522 | 2644658 | 2139522 | 1434050 | | 2644658 | 85133 | 101111 | 504209 | 101111 | 85133 | 504209 |
| **RR** | 0.67 | | 1.24 | | 0.54 | | | 0.84 | | 4.99 | | 0.17 | |
| **p-value** | 0.19 | | 0.63 | | 0.14 | | | 0.67 | | ***0.04*** | | ***0.02*** | |
|  | **mLN** | | | | | | | **BM** | | | | | |
| **CD45** |  | |  | |  | | |  | |  | |  | |
| **LSM** | 713220 | 500007 | 546103 | 500007 | 713220 | | 546103 | 458749 | 4618750 | 6246220 | 4618750 | 458749 | 6246220 |
| **RR** | 1.43 | | 1.09 | | 1.31 | | | 0.99 | | 1.35 | | 0.73 | |
| **p-value** | 0.35 | | 0.78 | | 0.43 | | | 0.98 | | 0.24 | | 0.24 | |
|  |  | | | | | | |  | | | | | |
| **CD3** |  | |  | |  | | |  | |  | |  | |
| **LSM** | 483107 | 313674 | 369278 | 313674 | 483107 | | 369278 | 758742 | 421653 | 1065456 | 421653 | 758742 | 1065456 |
| **RR** | 1.54 | | 1.18 | | 1.31 | | | 1.80 | | 2.53 | | 0.71 | |
| **p-value** | 0.23 | | 0.59 | | 0.40 | | | 0.21 | | 0.06 | | 0.60 | |
|  |  | | | | | | |  | | | | | |
| **CD4** |  | |  | |  | | |  | |  | |  | |
| **LSM** | 289881 | 162263 | 196322 | 162263 | 289881 | | 196322 | 491659 | 258899 | 750214 | 258899 | 491659 | 750214 |
| **RR** | 1.79 | | 1.21 | | 1.48 | | | 1.90 | | 2.90 | | 0.65 | |
| **p-value** | 0.11 | | 0.54 | | 0.22 | | | 0.23 | | 0.05 | | 0.56 | |
|  |  | | | | | | |  | | | | | |
| **CD8** |  | |  | |  | | |  | |  | |  | |
| **LSM** | 158481 | 117717 | 133753 | 117717 | 158481 | | 133753 | 137249 | 112557 | 222200 | 112557 | 137249 | 222200 |
| **RR** | 1.35 | | 1.14 | | 1.18 | | | 1.22 | | 1.97 | | 0.62 | |
| **p-value** | 0.43 | | 0.69 | | 0.59 | | | 0.60 | | 0.10 | | 0.33 | |
|  | **LI** | | | | | | | **SG** | | | | | |
| **CD45** |  | |  | |  | | |  | |  | |  | |
| **LSM** | 73558507 | 39174813 | 57231360 | 39174813 | 73558507 | | 57231360 | 193373 | 118040 | 311833 | 118040 | 193373 | 311833 |
| **RR** | 1.88 | | 1.46 | | 128 | | | 1.64 | | 2.64 | | 0.62 | |
| **p-value** | ***<0.01*** | | 0.09 | | 0.14 | | | 0.37 | | 0.24 | | 0.46 | |
|  |  | | | | | | |  | | | | | |
| **CD3** |  | |  | |  | | |  | |  | |  | |
| **LSM** | 29143525 | 23845641 | 25826715 | 23845641 | 29143525 | | 25826715 | 140195 | 69990 | 188816 | 69990 | 140195 | 188816 |
| **RR** | 1.22 | | 1.08 | | 1.13 | | | 2.00 | | 2.70 | | 0.74 | |
| **p-value** | 0.51 | | 0.81 | | 0.72 | | | 0.30 | | 0.29 | | 0.71 | |
|  |  | | | | | | |  | | | | | |
| **CD4** |  | |  | |  | | |  | |  | |  | |
| **LSM** | 20400095 | 17507023 | 17801154 | 17507023 | 20400095 | | 17801154 | 119325 | 32035 | 134466 | 32035 | 119325 | 134466 |
| **RR** | 1.16 | | 1.02 | | 1.15 | | | 3.72 | | 4.20 | | 0.89 | |
| **p-value** | 0.62 | | 0.96 | | 0.67 | | | ***0.04*** | | 0.10 | | 0.88 | |
|  |  | | | | | | |  | | | | | |
| **CD8** |  | |  | |  | | |  | |  | |  | |
| **LSM** | 6973181 | 5206818 | 6843448 | 5206818 | 6973181 | | 6843448 | 10361 | 24308 | 39863 | 24308 | 10361 | 39863 |
| **RR** | 1.34 | | 1.31 | | 1.02 | | | 0.43 | | 1.64 | | 0.26 | |
| **p-value** | 0.35 | | 0.34 | | 0.96 | | | 0.29 | | 0.63 | | 0.13 | |

**Supplementary Table 5: Descriptive statistics regarding analyses various organs for immune phenotype of NRG mice.** Note: LSM: least squares means estimation; RR: relative rate (between REAC and CTR; INF and CTR; REAC and INF).

| **PD-1** | **SPL** | | | | | | | **LN** | | | | | |
| --- | --- | --- | --- | --- | --- | --- | --- | --- | --- | --- | --- | --- | --- |
|  | **REAC** | **CTR** | **INF** | **CTR** | **REAC** | **INF** | | **REAC** | **CTR** | **INF** | **CTR** | **REAC** | **INF** |
| **CD3** |  | |  | |  | | |  | |  | |  | |
| **LSM** | 3317529 | 2632789 | 4447236 | 2632789 | 3317529 | | 4447236 | 175286 | 82157 | 116522 | 82157 | 175286 | 116522 |
| **RR** | 1.26 | | 1.69 | | 0.75 | | | 2.13 | | 1.42 | | 1.50 | |
| **p-value** | 0.53 | | 0.24 | | 0.56 | | | 0.17 | | 0.49 | | 0.39 | |
|  |  | | | | | | |  | | | | | |
| **CD4** |  | |  | |  | | |  | |  | |  | |
| **LSM** | 1677470 | 861540 | 1954880 | 861540 | 1677470 | | 1954880 | 120347 | 60444 | 78451 | 60444 | 120347 | 78451 |
| **RR** | 1.95 | | 2.27 | | 0.86 | | | 1.05 | | 1.30 | | 1.53 | |
| **p-value** | 0.15 | | 0.10 | | 0.81 | | | 0.30 | | 0.64 | | 0.47 | |
|  |  | | | | | | |  | | | | | |
| **CD8** |  | |  | |  | | |  | |  | |  | |
| **LSM** | 1174436 | 1412642 | 2124129 | 1412642 | 1174436 | | 2124129 | 35405 | 13188 | 26321 | 13188 | 35405 | 26321 |
| **RR** | 0.83 | | 1.50 | | 0.55 | | | 2.68 | | 1.99 | | 1.34 | |
| **p-value** | 0.59 | | 0.40 | | 0.18 | | | 0.09 | | 0.06 | | 0.57 | |
|  | **mLN** | | | | | | | **BM** | | | | | |
| **CD3** |  | |  | |  | | |  | |  | |  | |
| **LSM** | 272972 | 101002 | 153573 | 101002 | 272972 | | 153573 | 1091015 | 343675 | 501195 | 343675 | 1091015 | 501195 |
| **RR** | 2.70 | | 1.52 | | 1.78 | | | 3.17 | | 1.46 | | 2.18 | |
| **p-value** | ***0.04*** | | 0.06 | | 0.22 | | | ***0.03*** | | 0.10 | | 0.16 | |
|  |  | | | | | | |  | | | | | |
| **CD4** |  | |  | |  | | |  | |  | |  | |
| **LSM** | 195219 | 82445 | 104034 | 82445 | 195219 | | 104034 | 467957 | 226514 | 697991 | 226514 | 467957 | 697991 |
| **RR** | 2.37 | | 1.26 | | 1.88 | | | 2.06 | | 3.08 | | 0.67 | |
| **p-value** | 0.07 | | 0.21 | | 0.19 | | | 0.18 | | ***0.04*** | | 0.59 | |
|  |  | | | | | | |  | | | | | |
| **CD8** |  | |  | |  | | |  | |  | |  | |
| **LSM** | 66065 | 17385 | 40001 | 17385 | 66065 | | 40001 | 130571 | 93600 | 193143 | 93600 | 130571 | 193143 |
| **RR** | 3.80 | | 2.30 | | 1.65 | | | 1.39 | | 2.06 | | 0.68 | |
| **p-value** | ***<0.01*** | | ***<0.01*** | | 0.28 | | | 0.39 | | 0.08 | | 0.43 | |
|  | **LI** | | | | | | | **SG** | | | | | |
| **CD3** |  | |  | |  | | |  | |  | |  | |
| **LSM** | 17983265 | 11959975 | 14759365 | 11959975 | 17983265 | | 14759365 | 122677 | 22231 | 75266 | 2231 | 12677 | 75266 |
| **RR** | 1.50 | | 1.23 | | 1.22 | | | 3.72 | | 4.19 | | 0.89 | |
| **p-value** | 0.34 | | 0.63 | | 0.69 | | | **<0.01** | | 0.11 | | 0.86 | |
|  |  | | | | | | |  | | | | | |
| **CD4** |  | |  | |  | | |  | |  | |  | |
| **LSM** | 11486534 | 8744391 | 9814834 | 8744391 | 11486534 | | 9814834 | 109921 | 15372 | 62563 | 15372 | 109921 | 62563 |
| **RR** | 1.31 | | 1.12 | | 1.17 | | | 7.15 | | 4.07 | | 1.76 | |
| **p-value** | 0.54 | | 0.79 | | 0.75 | | | ***<0.01*** | | **0.04** | | 0.46 | |
|  |  | | | | | | |  | | | | | |
| **CD8** |  | |  | |  | | |  | |  | |  | |
| **LSM** | 46627592 | 16530838 | 27825436 | 16530838 | 46627592 | | 27825436 | 7604 | 22066 | 33121 | 22066 | 7604 | 33121 |
| **RR** | 2.82 | | 1.68 | | 1.67 | | | 0.34 | | 1.50 | | 0.23 | |
| **p-value** | ***0.01*** | | 0.24 | | 0.11 | | | 0.24 | | 0.71 | | 0.13 | |

**Supplementary Table 6: Descriptive statistics regarding analyses various organs for T cell phenotype and PD-1 expression of NRG mice.** Note: LSM: least squares means estimation; RR: relative rate (between REAC and CTR; INF and CTR; REAC and INF).

| **A) CD4** | **SPL** | | | | | | | **LN** | | | | | |
| --- | --- | --- | --- | --- | --- | --- | --- | --- | --- | --- | --- | --- | --- |
|  | **REAC** | **CTR** | **INF** | **CTR** | **REAC** | **INF** | | **REAC** | **CTR** | **INF** | **CTR** | **REAC** | **INF** |
| **N** |  | | | | | | |  | | | | | |
| **LSM** | 207660 | 262202 | 148464 | 262202 | 207660 | 148464 | | 26591 | 29478 | 23955 | 29478 | 26591 | 23955 |
| **RR** | 0.79 | | 0.57 | | 1.40 | | | 0.90 | | 0.81 | | 1.11 | |
| **p-value** | 0.62 | | 0.27 | | 0.52 | | | 0.84 | | 0.71 | | 0.80 | |
|  |  | | | | | | |  | | | | | |
| **CM** |  | |  | |  | | |  | |  | |  | |
| **LSM** | 201592 | 352080 | 227000 | 352080 | 201592 | | 227000 | 48029 | 70801 | 550953 | 70801 | 48029 | 550953 |
| **RR** | 0.57 | | 0.64 | | 0.89 | | | 0.68 | | 7.78 | | 0.09 | |
| **p-value** | 0.24 | | 0.11 | | 0.81 | | | 0.43 | | ***0.02*** | | ***<0.01*** | |
|  |  | | | | | | |  | | | | | |
| **EM** |  | |  | |  | | |  | |  | |  | |
| **LSM** | 685403 | 730529 | 1834088 | 730529 | 685403 | | 1834088 | 84410 | 54840 | 532670 | 54840 | 84410 | 532670 |
| **RR** | 0.94 | | 2.51 | | 0.37 | | | 1.54 | | 9.71 | | 0.16 | |
| **p-value** | 0.88 | | 0.12 | | 0.08 | | | 0.40 | | ***0.01*** | | ***0.04*** | |
|  |  | | | | | | |  | | | | | |
| **TE** |  | |  | |  | | |  | |  | |  | |
| **LSM** | 871427 | 97198 | 74620 | 97198 | 871427 | | 74620 | 22584 | 17507 | 22064 | 17507 | 22584 | 22064 |
| **RR** | 8.96 | | 0.77 | | 11.68 | | | 1.29 | | 1.26 | | 1.02 | |
| **p-value** | ***0.02*** | | 0.48 | | ***<0.01*** | | | 0.62 | | 0.63 | | 0.95 | |
|  | **mLN** | | | | | | | **BM** | | | | | |
| **N** |  | |  | |  | | |  | |  | |  | |
| **LSM** | 41613 | 27137 | 20186 | 27137 | 41613 | | 20186 | 29762 | 14209 | 10915 | 14209 | 29762 | 10915 |
| **RR** | 1.53 | | 0.74 | | 2.06 | | | 2.09 | | 0.77 | | 2.73 | |
| **p-value** | 0.40 | | 0.54 | | 0.13 | | | 0.41 | | 0.64 | | 0.27 | |
|  |  | | | | | | |  | | | | | |
| **CM** |  | |  | |  | | |  | |  | |  | |
| **LSM** | 101393 | 66996 | 59125 | 66996 | 101393 | | 59125 | 63290 | 89637 | 227692 | 89637 | 63290 | 227692 |
| **RR** | 1.51 | | 0.88 | | 1.71 | | | 0.71 | | 2.54 | | 0.28 | |
| **p-value** | 0.39 | | 0.73 | | 0.23 | | | 0.37 | | 0.20 | | 0.09 | |
|  |  | | | | | | |  | | | | | |
| **EM** |  | |  | |  | | |  | |  | |  | |
| **LSM** | 128144 | 57149 | 98686 | 57149 | 128144 | | 98686 | 142264 | 143317 | 496828 | 143317 | 142264 | 496828 |
| **RR** | 2.24 | | 1.73 | | 1.30 | | | 0.99 | | 3.47 | | 0.29 | |
| **p-value** | ***0.05*** | | 0.26 | | 0.51 | | | 0.98 | | ***0.02*** | | ***0.01*** | |
|  |  | | | | | | |  | | | | | |
| **TE** |  | |  | |  | | |  | |  | |  | |
| **LSM** | 18649 | 11040 | 18290 | 11040 | 18649 | | 18290 | 256483 | 11500 | 14831 | 11500 | 256483 | 14831 |
| **RR** | 1.69 | | 1.66 | | 1.02 | | | 22.30 | | 1.29 | | 17.29 | |
| **p-value** | 0.35 | | 0.37 | | 0.97 | | | ***< 0.01*** | | 0.57 | | ***< 0.01*** | |
|  | **LI** | | | | | | | **SG** | | | | | |
| **N** |  | |  | |  | | |  | |  | |  | |
| **LSM** | 4925239 | 6116871 | 3708883 | 6116871 | 4925239 | | 3708883 | 8726 | 14890 | 58910 | 14890 | 8726 | 58910 |
| **RR** | 0.80 | | 0.61 | | 1.33 | | | 0.59 | | 3.96 | | 0.15 | |
| **p-value** | 0.46 | | 0.15 | | 0.15 | | | 0.49 | | 0.21 | | ***0.03*** | |
|  |  | | | | | | |  | | | | | |
| **CM** |  | |  | |  | | |  | |  | |  | |
| **LSM** | 9735359 | 7469599 | 8281544 | 7469599 | 9735359 | | 8281544 | 16890 | 8585 | 47829 | 8585 | 16890 | 47829 |
| **RR** | 1.30 | | 1.11 | | 1.17 | | | 1.97 | | 5.57 | | 0.35 | |
| **p-value** | 0.50 | | 0.79 | | 0.71 | | | 0.43 | | 0.10 | | 0.22 | |
|  |  | | | | | | |  | | | | | |
| **EM** |  | |  | |  | | |  | |  | |  | |
| **LSM** | 4301431 | 2594159 | 4188082 | 2594159 | 4301431 | | 4188082 | 21057 | 4672 | 23522 | 4672 | 21057 | 23522 |
| **RR** | 1.66 | | 1.61 | | 1.03 | | | 4.50 | | 5.03 | | 0.89 | |
| **p-value** | 0.24 | | 0.28 | | 0.95 | | | ***0.02*** | | ***0.01*** | | 0.88 | |
|  |  | | | | | | |  | | | | | |
| **TE** |  | |  | |  | | |  | |  | |  | |
| **LSM** | 1456921 | 1323700 | 1628741 | 1323700 | 1456921 | | 1628741 | 72649 | 3899 | 4018 | 3899 | 72649 | 4018 |
| **RR** | 1.10 | | 1.23 | | 0.89 | | | 18.63 | | 1.03 | | 18.08 | |
| **p-value** | 0.79 | | 0.63 | | 0.75 | | | ***0.01*** | | 0.97 | | ***0.01*** | |

| **B) CD4 PD-1** | **SPL** | | | | | | | **LN** | | | | | |
| --- | --- | --- | --- | --- | --- | --- | --- | --- | --- | --- | --- | --- | --- |
|  | **REAC** | **CTR** | **INF** | **CTR** | **REAC** | **INF** | | **REAC** | **CTR** | **INF** | **CTR** | **REAC** | **INF** |
| **N** |  | | | | | | |  | | | | | |
| **LSM** | 83188 | 39258 | 148464 | 262202 | 207660 | 148464 | | 26591 | 29478 | 23955 | 29478 | 26591 | 23955 |
| **RR** | 2.12 | | 0.36 | | 1.40 | | | 0.90 | | 0.81 | | 1.11 | |
| **p-value** | 0.35 | | ***0.04*** | | 0.52 | | | 0.84 | | 0.71 | | 0.80 | |
|  |  | | | | | | |  | | | | | |
| **CM** |  | |  | |  | | |  | |  | |  | |
| **LSM** | 161296 | 270771 | 227000 | 352080 | 201592 | | 227000 | 48029 | 70801 | 550953 | 70801 | 48029 | 550953 |
| **RR** | 0.60 | | 0.62 | | 0.89 | | | 0.68 | | 7.78 | | 0.09 | |
| **p-value** | 0.31 | | 0.13 | | 0.81 | | | 0.43 | | ***0.02*** | | ***<0.01*** | |
|  |  | | | | | | |  | | | | | |
| **EM** |  | |  | |  | | |  | |  | |  | |
| **LSM** | 615427 | 573580 | 1834088 | 730529 | 685403 | | 1834088 | 84410 | 54840 | 532670 | 54840 | 84410 | 532670 |
| **RR** | 1.07 | | 2.92 | | 0.37 | | | 1.54 | | 9.71 | | 0.16 | |
| **p-value** | 0.86 | | 0.07 | | 0.08 | | | 0.40 | | ***0.01*** | | ***0.04*** | |
|  |  | | | | | | |  | | | | | |
| **TE** |  | |  | |  | | |  | |  | |  | |
| **LSM** | 807153 | 19295 | 74620 | 97198 | 871427 | | 74620 | 22584 | 17507 | 22064 | 17507 | 22584 | 22064 |
| **RR** | 41.83 | | 0.91 | | 11.68 | | | 1.29 | | 1.26 | | 1.02 | |
| **p-value** | ***<0.01*** | | 0.85 | | ***<0.01*** | | | 0.62 | | 0.63 | | 0.95 | |
|  | **mLN** | | | | | | | **BM** | | | | | |
| **N** |  | |  | |  | | |  | |  | |  | |
| **LSM** | 3034 | 5588 | 20186 | 27137 | 41613 | | 20186 | 29762 | 14209 | 10915 | 14209 | 29762 | 10915 |
| **RR** | 0.54 | | 0.32 | | 2.06 | | | 2.09 | | 0.77 | | 2.73 | |
| **p-value** | 0.14 | | ***<0.01*** | | 0.13 | | | 0.41 | | 0.64 | | 0.27 | |
|  |  | | | | | | |  | | | | | |
| **CM** |  | |  | |  | | |  | |  | |  | |
| **LSM** | 77576 | 47106 | 59125 | 66996 | 101393 | | 59125 | 63290 | 89637 | 227692 | 89637 | 63290 | 227692 |
| **RR** | 1.65 | | 1.04 | | 1.71 | | | 0.71 | | 2.54 | | 0.28 | |
| **p-value** | 0.45 | | 0.90 | | 0.23 | | | 0.37 | | 0.20 | | 0.09 | |
|  |  | | | | | | |  | | | | | |
| **EM** |  | |  | |  | | |  | |  | |  | |
| **LSM** | 108894 | 23378 | 98686 | 57149 | 128144 | | 98686 | 142264 | 143317 | 496828 | 143317 | 142264 | 496828 |
| **RR** | 4.66 | | 2.11 | | 1.30 | | | 0.99 | | 3.47 | | 0.29 | |
| **p-value** | ***<0.01*** | | ***<0.01*** | | 0.51 | | | 0.98 | | ***0.02*** | | ***0.01*** | |
|  |  | | | | | | |  | | | | | |
| **TE** |  | |  | |  | | |  | |  | |  | |
| **LSM** | 3115 | 1505 | 18290 | 11040 | 18649 | | 18290 | 256483 | 11500 | 14831 | 11500 | 256483 | 14831 |
| **RR** | 2.07 | | 0.90 | | 1.02 | | | 22.30 | | 1.29 | | 17.29 | |
| **p-value** | 0.17 | | 0.73 | | 0.97 | | | ***< 0.01*** | | 0.57 | | ***< 0.01*** | |
|  | **LI** | | | | | | | **SG** | | | | | |
| **N** |  | |  | |  | | |  | |  | |  | |
| **LSM** | 332040 | 721180 | 3708883 | 6116871 | 4925239 | | 3708883 | 8726 | 14890 | 58910 | 14890 | 8726 | 58910 |
| **RR** | 0.46 | | 0.18 | | 1.33 | | | 0.59 | | 3.96 | | 0.15 | |
| **p-value** | 0.17 | | ***<0.01*** | | 0.15 | | | 0.49 | | 0.21 | | ***0.03*** | |
|  |  | | | | | | |  | | | | | |
| **CM** |  | |  | |  | | |  | |  | |  | |
| **LSM** | 8072127 | 6024800 | 8281544 | 7469599 | 9735359 | | 8281544 | 16890 | 8585 | 47829 | 8585 | 16890 | 47829 |
| **RR** | 1.34 | | 1.13 | | 1.17 | | | 1.97 | | 5.57 | | 0.35 | |
| **p-value** | 0.50 | | 0.78 | | 0.71 | | | 0.43 | | 0.10 | | 0.22 | |
|  |  | | | | | | |  | | | | | |
| **EM** |  | |  | |  | | |  | |  | |  | |
| **LSM** | 3687858 | 1958544 | 4188082 | 2594159 | 4301431 | | 4188082 | 21057 | 4672 | 23522 | 4672 | 21057 | 23522 |
| **RR** | 1.88 | | 1.79 | | 1.03 | | | 4.50 | | 5.03 | | 0.89 | |
| **p-value** | 0.17 | | 0.21 | | 0.95 | | | ***0.02*** | | ***0.01*** | | 0.88 | |
|  |  | | | | | | |  | | | | | |
| **TE** |  | |  | |  | | |  | |  | |  | |
| **LSM** | 82153 | 183628 | 1628741 | 1323700 | 1456921 | | 1628741 | 72649 | 3899 | 4018 | 3899 | 72649 | 4018 |
| **RR** | 0.45 | | 0.19 | | 0.89 | | | 18.63 | | 1.03 | | 18.08 | |
| **p-value** | 0.13 | | ***0.01*** | | 0.75 | | | ***0.01*** | | 0.97 | | ***0.01*** | |

| **C) CD8** | **SPL** | | | | | | | **LN** | | | | | |
| --- | --- | --- | --- | --- | --- | --- | --- | --- | --- | --- | --- | --- | --- |
|  | **REAC** | **CTR** | **INF** | **CTR** | **REAC** | **INF** | | **REAC** | **CTR** | **INF** | **CTR** | **REAC** | **INF** |
| **N** |  | | | | | | |  | | | | | |
| **LSM** | 121813 | 257356 | 144649 | 257356 | 121813 | 144649 | | 13988 | 26911 | 27709 | 26911 | 13988 | 27709 |
| **RR** | 0.47 | | 0.56 | | 0.84 | | | 0.52 | | 1.03 | | 0.50 | |
| **p-value** | 0.14 | | 0.19 | | 0.76 | | | 0.20 | | 0.96 | | 0.20 | |
|  |  | | | | | | |  | | | | | |
| **CM** |  | |  | |  | | |  | |  | |  | |
| **LSM** | 393422 | 576377 | 728479 | 576377 | 393422 | | 728479 | 20224 | 32399 | 176782 | 32399 | 20224 | 176782 |
| **RR** | 0.68 | | 1.26 | | 0.54 | | | 0.62 | | 5.46 | | 0.11 | |
| **p-value** | 0.24 | | 0.61 | | 0.12 | | | 0.40 | | 0.05 | | ***0.01*** | |
|  |  | | | | | | |  | | | | | |
| **EM** |  | |  | |  | | |  | |  | |  | |
| **LSM** | 874916 | 1210139 | 1733976 | 1210139 | 874916 | | 1733976 | 36521 | 28008 | 248863 | 28008 | 36521 | 248863 |
| **RR** | 0.72 | | 1.43 | | 0.50 | | | 1.30 | | 8.88 | | 0.15 | |
| **p-value** | 0.47 | | ***0.05*** | | 0.20 | | | 0.61 | | ***0.01*** | | 0.02 | |
|  |  | | | | | | |  | | | | | |
| **TE** |  | |  | |  | | |  | |  | |  | |
| **LSM** | 44416 | 97815 | 33880 | 97815 | 44416 | | 33880 | 14401 | 13760 | 48777 | 13760 | 14401 | 48777 |
| **RR** | 0.45 | | 0.35 | | 1.31 | | | 1.05 | | 3.54 | | 0.29 | |
| **p-value** | 0.31 | | ***0.02*** | | 0.43 | | | 0.92 | | 0.14 | | 0.13 | |
|  | **mLN** | | | | | | | **BM** | | | | | |
| **N** |  | |  | |  | | |  | |  | |  | |
| **LSM** | 43733 | 44385 | 31840 | 44385 | 43733 | | 31840 | 3169 | 13210 | 6702 | 13210 | 3169 | 6702 |
| **RR** | 0.98 | | 0.72 | | 1.37 | | | 0.24 | | 0.51 | | 0.47 | |
| **p-value** | 0.98 | | 0.54 | | 0.53 | | | ***0.01*** | | 0.18 | | 0.09 | |
|  |  | | | | | | |  | | | | | |
| **CM** |  | |  | |  | | |  | |  | |  | |
| **LSM** | 36739 | 24753 | 29807 | 24753 | 36739 | | 29807 | 11711 | 24389 | 38660 | 24389 | 11711 | 38660 |
| **RR** | 1.48 | | 1.20 | | 1.23 | | | 0.48 | | 1.58 | | 0.30 | |
| **p-value** | 0.40 | | 0.69 | | 0.65 | | | 0.07 | | 0.51 | | 0.11 | |
|  |  | | | | | | |  | | | | | |
| **EM** |  | |  | |  | | |  | |  | |  | |
| **LSM** | 52411 | 35826 | 48642 | 35826 | 52411 | | 48642 | 81244 | 63071 | 147218 | 63071 | 81244 | 147218 |
| **RR** | 1.46 | | 1.36 | | 1.08 | | | 1.29 | | 2.33 | | 0.55 | |
| **p-value** | 0.52 | | 0.61 | | 0.87 | | | 0.55 | | 0.07 | | 0.25 | |
|  |  | | | | | | |  | | | | | |
| **TE** |  | |  | |  | | |  | |  | |  | |
| **LSM** | 25512 | 12849 | 23445 | 12849 | 25512 | | 23445 | 41114 | 11805 | 29495 | 11805 | 41114 | 29495 |
| **RR** | 1.98 | | 1.82 | | 1.09 | | | 3.48 | | 2.50 | | 1.39 | |
| **p-value** | 0.06 | | 0.14 | | 0.83 | | | 0.06 | | 0.18 | | 0.68 | |
|  | **LI** | | | | | | | **SG** | | | | | |
| **N** |  | |  | |  | | |  | |  | |  | |
| **LSM** | 1537594 | 2635449 | 1670210 | 2635449 | 1537594 | | 1670210 | 2395 | 18292 | 26235 | 18292 | 2395 | 26235 |
| **RR** | 0.58 | | 0.63 | | 0.92 | | | 0.13 | | 1.43 | | 0.09 | |
| **p-value** | ***0.05*** | | 0.10 | | 0.72 | | | ***0.02*** | | 0.75 | | ***0.01*** | |
|  |  | | | | | | |  | | | | | |
| **CM** |  | |  | |  | | |  | |  | |  | |
| **LSM** | 3249423 | 1446866 | 3084622 | 1446866 | 3249423 | | 3084622 | 5208 | 3946 | 9077 | 3946 | 5208 | 9077 |
| **RR** | 2.24 | | 2.13 | | 1.05 | | | 1.32 | | 2.30 | | 0.57 | |
| **p-value** | 0.11 | | 0.06 | | 0.92 | | | 0.73 | | 0.36 | | 0.57 | |
|  |  | | | | | | |  | | | | | |
| **EM** |  | |  | |  | | |  | |  | |  | |
| **LSM** | 1435636 | 555406 | 1422601 | 555406 | 1435636 | | 1422601 | 2334 | 1165 | 3524 | 1165 | 2334 | 3524 |
| **RR** | 2.58 | | 2.56 | | 1.01 | | | 2.00 | | 3.02 | | 0.66 | |
| **p-value** | ***0.01*** | | 0.06 | | 0.98 | | | 0.22 | | ***0.02*** | | 0.56 | |
|  |  | | | | | | |  | | | | | |
| **TE** |  | |  | |  | | |  | |  | |  | |
| **LSM** | 5179846 | 1700462 | 2224851 | 1700462 | 5179846 | | 2224851 | 408 | 925 | 1114 | 925 | 408 | 1114 |
| **RR** | 3.05 | | 1.31 | | 2.33 | | | 0.44 | | 1.20 | | 0.37 | |
| **p-value** | ***0.01*** | | 0.43 | | ***0.02*** | | | 0.21 | | 0.82 | | 0.25 | |

| **D) CD8 PD-1** | **SPL** | | | | | | | **LN** | | | | | |
| --- | --- | --- | --- | --- | --- | --- | --- | --- | --- | --- | --- | --- | --- |
|  | **REAC** | **CTR** | **INF** | **CTR** | **REAC** | **INF** | | **REAC** | **CTR** | **INF** | **CTR** | **REAC** | **INF** |
| **N** |  | | | | | | |  | | | | | |
| **LSM** | 44254 | 60566 | 29826 | 60566 | 44254 | 29826 | | 5954 | 5720 | 3042 | 5720 | 5954 | 3042 |
| **RR** | 0.73 | | 0.49 | | 1.48 | | | 1.04 | | 0.53 | | 1.96 | |
| **p-value** | 0.65 | | 0.08 | | 0.55 | | | 0.95 | | 0.23 | | 0.26 | |
|  |  | | | | | | |  | | | | | |
| **CM** |  | |  | |  | | |  | |  | |  | |
| **LSM** | 328917 | 352567 | 541487 | 352567 | 328917 | | 541487 | 48099 | 58628 | 36362 | 58628 | 48099 | 36362 |
| **RR** | 0.93 | | 1.54 | | 0.61 | | | 0.82 | | 0.62 | | 1.32 | |
| **p-value** | 0.83 | | 0.36 | | 0.25 | | | 0.71 | | 0.25 | | 0.46 | |
|  |  | | | | | | |  | | | | | |
| **EM** |  | |  | |  | | |  | |  | |  | |
| **LSM** | 748579 | 829255 | 1441270 | 829255 | 748579 | | 1441270 | 22261 | 5251 | 20966 | 5251 | 22261 | 20966 |
| **RR** | 0.90 | | 1.74 | | 0.52 | | | 4.24 | | 3.99 | | 1.06 | |
| **p-value** | 0.82 | | 0.31 | | 0.22 | | | ***<0.01*** | | ***<0.01*** | | 0.86 | |
|  |  | | | | | | |  | | | | | |
| **TE** |  | |  | |  | | |  | |  | |  | |
| **LSM** | 20283 | 29903 | 12788 | 29903 | 20283 | | 12788 | 5954 | 2790 | 1221 | 2790 | 5954 | 1221 |
| **RR** | 0.68 | | 0.43 | | 1.59 | | | 2.13 | | 0.44 | | 4.88 | |
| **p-value** | 0.61 | | 0.08 | | 0.48 | | | 0.24 | | ***0.03*** | | ***0.01*** | |
|  | **mLN** | | | | | | | **BM** | | | | | |
| **N** |  | |  | |  | | |  | |  | |  | |
| **LSM** | 15888 | 20644 | 9121 | 20644 | 15888 | | 9121 | 2002 | 9811 | 2267 | 9811 | 2002 | 2267 |
| **RR** | 0.77 | | 0.44 | | 1.74 | | | 0.20 | | 0.23 | | 0.88 | |
| **p-value** | 0.57 | | ***<0.01*** | | 0.24 | | | ***< 0.01*** | | ***< 0.01*** | | 0.83 | |
|  |  | | | | | | |  | | | | | |
| **CM** |  | |  | |  | | |  | |  | |  | |
| **LSM** | 27866 | 13131 | 20055 | 13131 | 27866 | | 20055 | 1980 | 7917 | 2251 | 7917 | 1980 | 2251 |
| **RR** | 2.12 | | 1.53 | | 1.39 | | | 0.25 | | 0.28 | | 0.88 | |
| **p-value** | 0.11 | | 0.17 | | 0.55 | | | ***0.02*** | | ***0.02*** | | 0.87 | |
|  |  | | | | | | |  | | | | | |
| **EM** |  | |  | |  | | |  | |  | |  | |
| **LSM** | 14152 | 5033 | 16797 | 5033 | 14152 | | 16797 | 112139 | 65666 | 115642 | 65666 | 112139 | 115642 |
| **RR** | 2.81 | | 3.34 | | 0.84 | | | 1.71 | | 1.76 | | 0.97 | |
| **p-value** | ***0.04*** | | ***<0.01*** | | 0.74 | | | 0.16 | | 0.23 | | 0.96 | |
|  |  | | | | | | |  | | | | | |
| **TE** |  | |  | |  | | |  | |  | |  | |
| **LSM** | 6861 | 3063 | 3387 | 3063 | 6861 | | 3387 | 75118 | 12613 | 34088 | 12613 | 75118 | 34088 |
| **RR** | 2.24 | | 1.11 | | 2.02 | | | 5.95 | | 2.70 | | 2.20 | |
| **p-value** | 0.16 | | 0.60 | | 0.22 | | | ***< 0.01*** | | 0.17 | | 0.35 | |
|  | **LI** | | | | | | | **SG** | | | | | |
| **N** |  | |  | |  | | |  | |  | |  | |
| **LSM** | 374941 | 391338 | 98639 | 391338 | 374941 | | 98639 | 525 | 704 | 1201 | 704 | 525 | 1201 |
| **RR** | 0.96 | | 0.25 | | 3.80 | | | 0.75 | | 1.71 | | 0.44 | |
| **p-value** | 0.92 | | ***<0.01*** | | ***<0.01*** | | | 0.75 | | ***0.05*** | | 0.39 | |
|  |  | | | | | | |  | | | | | |
| **CM** |  | |  | |  | | |  | |  | |  | |
| **LSM** | 2771827 | 1157516 | 2282670 | 1157516 | 2771827 | | 2282670 | 2455 | 1372 | 3832 | 1372 | 2455 | 3832 |
| **RR** | 2.39 | | 1.97 | | 1.21 | | | 1.79 | | 2.79 | | 0.64 | |
| **p-value** | 0.12 | | 0.13 | | 0.75 | | | 0.40 | | 0.22 | | 0.63 | |
|  |  | | | | | | |  | | | | | |
| **EM** |  | |  | |  | | |  | |  | |  | |
| **LSM** | 1274155 | 484417 | 1193367 | 484417 | 1274155 | | 1193367 | 1635 | 638 | 2279 | 638 | 1635 | 2279 |
| **RR** | 2.63 | | 2.46 | | 1.07 | | | 2.56 | | 3.57 | | 0.72 | |
| **p-value** | ***0.01*** | | 0.10 | | 0.90 | | | 0.16 | | ***0.03*** | | 0.61 | |
|  |  | | | | | | |  | | | | | |
| **TE** |  | |  | |  | | |  | |  | |  | |
| **LSM** | 5179846 | 1700462 | 2224851 | 1700462 | 5179846 | | 2224851 | N.D. | N.D. | N.D. | N.D. | N.D. | N.D. |
| **RR** | 3.05 | | 1.31 | | 2.33 | | | N.D. | | N.D. | | N.D. | |
| **p-value** | ***0.01*** | | 0.43 | | ***0.02*** | | | N.D. | | N.D. | | N.D. | |

**Supplementary Table 7: Descriptive statistics regarding B cell analysis in NRG mice.** Note: LSM: least squares means estimation; RR: relative rate (between REAC and CTR; INF and CTR; REAC and INF).

| **A)** | **BM** | | | | | | | **SPL** | | | | | |
| --- | --- | --- | --- | --- | --- | --- | --- | --- | --- | --- | --- | --- | --- |
|  | **REAC** | **CTR** | **INF** | **CTR** | **REAC** | **INF** | | **REAC** | **CTR** | **INF** | **CTR** | **REAC** | **INF** |
| **T1** |  | | | | | | |  | | | | | |
| **LSM** | 6463988 | 6678210 | 8794436 | 6678210 | 6463988 | 8794436 | | 2047318 | 787763 | 849171 | 787763 | 2047318 | 849171 |
| **RR** | 0.97 | | 1.32 | | 0.73 | | | 2.60 | | 1.08 | | 2.41 | |
| **p-value** | 0.93 | | 0.45 | | 0.15 | | | ***0.01*** | | 0.84 | | ***0.01*** | |
|  |  | | | | | | |  | | | | | |
| **T2** |  | |  | |  | | |  | |  | |  | |
| **LSM** | 158731 | 166161 | 231622 | 166161 | 158731 | | 231622 | 1126165 | 565483 | 775275 | 565483 | 1126165 | 775275 |
| **RR** | 0.95 | | 1.39 | | 0.68 | | | 1.99 | | 1.37 | | 1.45 | |
| **p-value** | 0.90 | | 0.33 | | 0.22 | | | ***0.03*** | | 0.40 | | 0.29 | |
|  |  | | | | | | |  | | | | | |
| **MNBC** |  | |  | |  | | |  | |  | |  | |
| **LSM** | 142933 | 253832 | 310908 | 253832 | 142933 | | 310908 | 2114723 | 2485429 | 2157080 | 2485429 | 2114723 | 2157080 |
| **RR** | 0.56 | | 1.22 | | 0.46 | | | 0.85 | | 0.87 | | 0.98 | |
| **p-value** | 0.09 | | 0.54 | | ***0.03*** | | | 0.65 | | 0.66 | | 0.94 | |
|  |  | | | | | | |  | | | | | |
| **Pre-mBC** |  | |  | |  | | |  | |  | |  | |
| **LSM** | 30146 | 44923 | 49700 | 44923 | 30146 | | 49700 | 307934 | 219371 | 212172 | 219371 | 307934 | 212172 |
| **RR** | 0.67 | | 1.11 | | 0.61 | | | 1.40 | | 0.97 | | 1.45 | |
| **p-value** | 0.15 | | 0.66 | | 0.08 | | | 0.42 | | 0.94 | | 0.48 | |
|  | **LN** | | | | | | | **mLN** | | | | | |
| **T1** |  | |  | |  | | |  | |  | |  | |
| **LSM** | 777 | 45 | 179 | 45 | 777 | | 179 | 1229 | 404 | 490 | 404 | 1229 | 490 |
| **RR** | 17.26 | | 3.98 | | 4.33 | | | 3.04 | | 1.21 | | 2.51 | |
| **p-value** | ***<0.01*** | | ***0.02*** | | ***<0.01*** | | | 0.17 | | 0.75 | | 0.11 | |
|  |  | | | | | | |  | | | | | |
| **T2** |  | |  | |  | | |  | |  | |  | |
| **LSM** | 1803 | 473 | 1164 | 473 | 1803 | | 1164 | 16072 | 5210 | 8138 | 5210 | 16072 | 8138 |
| **RR** | 1.55 | | 3.81 | | 1.55 | | | 3.08 | | 1.56 | | 1.97 | |
| **p-value** | 0.32 | | 0.07 | | 0.32 | | | ***0.01*** | | 0.07 | | 0.13 | |
|  |  | | | | | | |  | | | | | |
| **MNBC** |  | |  | |  | | |  | |  | |  | |
| **LSM** | 86777 | 64023 | 53001 | 64023 | 86777 | | 53001 | 161227 | 192992 | 131403 | 192992 | 161227 | 131403 |
| **RR** | 1.35 | | 0.83 | | 1.64 | | | 0.83 | | 0.68 | | 1.23 | |
| **p-value** | 0.70 | | 0.81 | | 0.27 | | | 0.67 | | ***0.04*** | | 0.62 | |
|  |  | | | | | | |  | | | | | |
| **Pre-mBC** |  | |  | |  | | |  | |  | |  | |
| **LSM** | 2942 | 2032 | 1256 | 2032 | 2942 | | 1256 | 13945 | 13987 | 5964 | 13987 | 13945 | 5964 |
| **RR** | 1.45 | | 0.62 | | 2.34 | | | 1.00 | | 0.43 | | 2.33 | |
| **p-value** | 0.63 | | 0.53 | | ***0.03*** | | | 0.99 | | ***<0.01*** | | 0.09 | |
|  | **LI** | | | | | | |  |  |  |  |  |  |
| **T1** |  | |  | |  | | |  |  |  |  |  |  |
| **LSM** | 12114933 | 1678238 | 5662712 | 1678238 | 12114933 | | 5662712 |  |  |  |  |  |  |
| **RR** | 7.22 | | 3.37 | | 2.14 | | |  |  |  |  |  |  |
| **p-value** | ***<0.01*** | | ***<0.01*** | | ***<0.01*** | | |  |  |  |  |  |  |
|  |  | | | | | | |  |  |  |  |  |  |
| **T2** |  | |  | |  | | |  |  |  |  |  |  |
| **LSM** | 3017331 | 936233 | 2439340 | 936233 | 3017331 | | 2439340 |  |  |  |  |  |  |
| **RR** | 3.22 | | 2.60 | | 1.24 | | |  |  |  |  |  |  |
| **p-value** | ***<0.01*** | | ***<0.01*** | | 0.29 | | |  |  |  |  |  |  |
|  |  | | | | | | |  |  |  |  |  |  |
| **MNBC** |  | |  | |  | | |  |  |  |  |  |  |
| **LSM** | 7517442 | 3739309 | 6287311 | 3739309 | 7517442 | | 6287311 |  |  |  |  |  |  |
| **RR** | 2.01 | | 1.68 | | 1.20 | | |  |  |  |  |  |  |
| **p-value** | ***<0.01*** | | ***0.01*** | | 0.39 | | |  |  |  |  |  |  |
|  |  | | | | | | |  |  |  |  |  |  |
| **Pre-mBC** |  | |  | |  | | |  |  |  |  |  |  |
| **LSM** | 413686 | 227130 | 368519 | 227130 | 413686 | | 368519 |  |  |  |  |  |  |
| **RR** | 1.82 | | 1.62 | | 1.12 | | |  |  |  |  |  |  |
| **p-value** | ***<0.01*** | | ***<0.01*** | | 0.45 | | |  |  |  |  |  |  |

| **B)** | **BM** | | | | | | | **SPL** | | | | | |
| --- | --- | --- | --- | --- | --- | --- | --- | --- | --- | --- | --- | --- | --- |
|  | **REAC** | **CTR** | **INF** | **CTR** | **REAC** | **INF** | | **REAC** | **CTR** | **INF** | **CTR** | **REAC** | **INF** |
| **PB** |  | | | | | | |  | | | | | |
| **LSM** | 39180 | 45702 | 57522 | 45702 | 39180 | 57522 | | 169254 | 52126 | 47361 | 52126 | 169254 | 47361 |
| **RR** | 0.86 | | 1.26 | | 0.68 | | | 3.25 | | 0.91 | | 3.57 | |
| **p-value** | 0.65 | | 0.49 | | ***0.02*** | | | ***0.03*** | | 0.73 | | ***0.01*** | |
|  |  | | | | | | |  | | | | | |
| **PC** |  | |  | |  | | |  | |  | |  | |
| **LSM** | 931 | 1709 | 1078 | 1709 | 931 | | 1078 | 14657 | 5826 | 9499 | 5826 | 14657 | 9499 |
| **RR** | 0.54 | | 0.63 | | 0.86 | | | 2.52 | | 1.63 | | 1.54 | |
| **p-value** | ***0.03*** | | 0.49 | | 0.82 | | | 0.20 | | 0.54 | | 0.56 | |
|  |  | | | | | | |  | | | | | |
| **IgMmBC** |  | |  | |  | | |  | |  | |  | |
| **LSM** | 26179 | 36552 | 37187 | 36552 | 26179 | | 37187 | 224960 | 142249 | 145606 | 142249 | 224960 | 145606 |
| **RR** | 0.72 | | 1.02 | | 0.70 | | | 1.58 | | 1.02 | | 1.54 | |
| **p-value** | 0.35 | | 0.94 | | 0.25 | | | 0.14 | | 0.95 | | 0.15 | |
|  |  | | | | | | |  | | | | | |
| **IgGmBC** |  | |  | |  | | |  | |  | |  | |
| **LSM** | 1642 | 477 | 1429 | 477 | 1642 | | 1429 | 13464 | 2942 | 5747 | 2942 | 13464 | 5747 |
| **RR** | 3.44 | | 2.99 | | 1.15 | | | 4.58 | | 1.95 | | 2.34 | |
| **p-value** | 0.07 | | 0.11 | | 0.81 | | | ***0.01*** | | 0.42 | | 0.30 | |
|  | **LN** | | | | | | | **mLN** | | | | | |
| **PB** |  | |  | |  | | |  | |  | |  | |
| **LSM** | 34451 | 2851 | 3460 | 2851 | 34451 | | 3460 | 107740 | 13515 | 9738 | 13515 | 107740 | 9738 |
| **RR** | 12.08 | | 1.21 | | 9.96 | | | 7.97 | | 0.72 | | 11.06 | |
| **p-value** | ***<0.01*** | | 0.74 | | ***<0.01*** | | | ***<0.01*** | | 0.15 | | ***<0.01*** | |
|  |  | | | | | | |  | | | | | |
| **PC** |  | |  | |  | | |  | |  | |  | |
| **LSM** | 522 | 85 | 50 | 85 | 522 | | 50 | 2374 | 36 | 1358 | 36 | 2374 | 1358 |
| **RR** | 6.17 | | 0.59 | | 10.39 | | | 65.96 | | 37.73 | | 1.75 | |
| **p-value** | ***0.06*** | | 0.56 | | ***0.03*** | | | ***<0.01*** | | ***<0.01*** | | 0.58 | |
|  |  | | | | | | |  | | | | | |
| **IgMmBC** |  | |  | |  | | |  | |  | |  | |
| **LSM** | 11336 | 1535 | 2111 | 1535 | 11336 | | 2111 | 12619 | 5892 | 4471 | 5892 | 12619 | 4471 |
| **RR** | 7.39 | | 1.37 | | 5.37 | | | 2.14 | | 0.76 | | 2.82 | |
| **p-value** | ***0.02*** | | 0.69 | | ***0.01*** | | | 0.14 | | 0.28 | | 0.06 | |
|  |  | | | | | | |  | | | | | |
| **IgGmBC** |  | |  | |  | | |  | |  | |  | |
| **LSM** | 121 | 35 | 2 | 35 | 121 | | 2 | 796 | 41 | 244 | 41 | 796 | 244 |
| **RR** | 3.50 | | 0.05 | | 69.28 | | | 19.43 | | 5.96 | | 3.26 | |
| **p-value** | 0.26 | | ***0.01*** | | ***<0.01*** | | | ***<0.01*** | | 0.08 | | 0.17 | |
|  | **LI** | | | | | | |  |  |  |  |  |  |
| **PB** |  | |  | |  | | |  |  |  |  |  |  |
| **LSM** | 1228858 | 332943 | 764121 | 332943 | 1228858 | | 764121 |  |  |  |  |  |  |
| **RR** | 3.69 | | 2.29 | | 1.61 | | |  |  |  |  |  |  |
| **p-value** | ***<0.01*** | | ***0.02*** | | 0.25 | | |  |  |  |  |  |  |
|  |  | | | | | | |  |  |  |  |  |  |
| **PC** |  | |  | |  | | |  |  |  |  |  |  |
| **LSM** | 45827 | 18738 | 39143 | 18738 | 45827 | | 39143 |  |  |  |  |  |  |
| **RR** | 2.44 | | 2.09 | | 1.17 | | |  |  |  |  |  |  |
| **p-value** | *<0.01* | | *0.01* | | 0.55 | | |  |  |  |  |  |  |
|  |  | | | | | | |  |  |  |  |  |  |
| **IgMmBC** |  | |  | |  | | |  |  |  |  |  |  |
| **LSM** | 700593 | 291110 | 447751 | 291110 | 700593 | | 447751 |  |  |  |  |  |  |
| **RR** | 2.41 | | 1.54 | | 1.56 | | |  |  |  |  |  |  |
| **p-value** | ***<0.01*** | | 0.11 | | ***0.03*** | | |  |  |  |  |  |  |
|  |  | | | | | | |  |  |  |  |  |  |
| **IgGmBC** |  | |  | |  | | |  |  |  |  |  |  |
| **LSM** | N.D. | N.D. | N.D. | N.D. | N.D. | | N.D. |  |  |  |  |  |  |
| **RR** | N.D. | | N.D. | | N.D. | | |  |  |  |  |  |  |
| **p-value** | N.D. | | N.D. | | N.D. | | |  |  |  |  |  |  |

**Supplementary Table 8: Descriptive statistics regarding analyses of human antibodies detected in plasma.** LSM: least squares means estimation; MD: difference of the means (between CTR and INF; CTR and REAC; INF and REAC).

|  | **CTR** | **INF** | **CTR** | **REAC** | **INF** | **REAC** |
| --- | --- | --- | --- | --- | --- | --- |
| **IgG** |  | | | | | |
| LSM | 15.37 | 6.44 | 15.37 | 27.89 | 6.44 | 27.89 |
| MD | 8.93 | | -12.51 | | -21.45 | |
| p-value^1^ | 0.55 | | 0.40 | | 0.16 | |
|  |  | |  | |  | |
| **IgM** |  | | | | | |
| LSM | 52.91 | 49.85 | 52.91 | 74.67 | 49.85 | 74.67 |
| MD | 3.05 | | -21.76 | | -24.81 | |
| p-value | 0.91 | | 0.43 | | 0.37 | |
